# Chemogenetic control of GABAergic neurons within the interpeduncular nucleus reveals dissociable behavioral components of the nicotine withdrawal phenotype

**DOI:** 10.1016/j.nbd.2025.107240

**Published:** 2025-12-22

**Authors:** Anabel M.M. Miguelez Fernández, Shana Netherton, Seshadri B. Niladhuri, Alexander C. Brown, Patricia Rivera, Kuei Y. Tseng, Christian J. Peters

**Affiliations:** Department of Anatomy and Cell Biology, University of Illinois Chicago – College of Medicine, Chicago, IL 60612, USA

## Abstract

Chronic exposure to nicotine results in the development of a dependent state such that a withdrawal syndrome is elicited upon cessation of nicotine. The interpeduncular nucleus (IPN) contains a high concentration of nicotinic acetylcholine receptors (nAChRs) and has been identified as a key brain region involved in nicotine withdrawal. Here we investigated the contribution of two distinct subpopulations of IPN GABAergic neurons to nicotine withdrawal behaviors. Withdrawal was induced in mice by implantation of osmotic pumps containing nicotine, followed by precipitation by intraperitoneal injections of mecamylamine. Using a chemogenetic approach to specifically target Amigo1-expressing or Epyc-expressing neurons within the IPN, we found that activity of the Amigo1 subpopulation of GABAergic neurons is critical for anxiety-like behaviors both in naïve mice and in those undergoing nicotine withdrawal. Moreover, data revealed that stimulation of Amigo1 neurons in nicotine-naïve mice elicits opposite effects on affective and somatic behaviors. Taken together, these results suggest that somatic and affective behaviors constitute dissociable components of the nicotine withdrawal phenotype and are likely supported by distinct subpopulations of neurons within the IPN.

## 1. Introduction

Chronic exposure to nicotine is known to saturate neuronal isoforms of nicotinic acetylcholine receptors (Brody et al., 2006), which become overresponsive during periods of abstinence, underpinning the expression of withdrawal symptoms. In humans, nicotine withdrawal is typically associated with affective symptoms such as heightened anxiety, depression, irritability and anhedonia (Association, A. P, 2022; Hughes, 2006, 2007), along with somatic symptoms, including increased appetite, insomnia and restlessness (Association, A. P, 2022; Hughes, 2007), the alleviation of which underlies the drive to continue smoking. While targeting neutralization of nicotine withdrawal represents an important component of nicotine cessation strategies, effective exploitation of this approach will require a more complete understanding of key neural mechanisms contributing to withdrawal from chronic nicotine exposure.

Nicotine withdrawal can be effectively modeled in rodents, whose behavioral changes recapitulate many of the symptoms of nicotine withdrawal in humans (McLaughlin et al., 2015). The most common behavioral observations associated with nicotine withdrawal in mice are increases in stereotypic behaviors indicative of physical discomfort reflecting a rodent somatic phenotype, and affective manifestations including signs of anhedonia, depression, hyperalgesia and anxiety, as reflected in well-validated behavior tasks (Chellian et al., 2021; McLaughlin et al., 2015). Spontaneous nicotine withdrawal in mice occurs within 24 h of removal of the nicotine source, and systemic injection of the nAChR antagonist mecamylamine can precipitate a similar withdrawal phenotype (Damaj et al., 2003; Malin et al., 1994). Direct injection of mecamylamine to the interpeduncular nucleus (IPN) is sufficient to phenocopy the somatic (Salas et al., 2009) or affective (Zhao-Shea et al., 2015) signs of withdrawal in nicotine-exposed animals, underscoring the contribution of the IPN to the nicotine withdrawal phenotype. The IPN is a GABAergic nucleus in the ventral midbrain, which receives its primary afferent input from the medial habenula and projects to the mesopontine Raphe and laterodorsal tegmentum (Lima et al., 2017; Quina et al., 2017). Knockout of α5 nAChR subunits in IPN suppresses somatic signs (Jackson et al., 2008), and optogenetic silencing of GAD2+ IPN neurons reduces somatic and affective signs in animals undergoing nicotine withdrawal (Klenowski et al., 2022). Taken together, these results suggest that dynamic changes in IPN neuronal activity following chronic nicotine exposure could drive the behavioral manifestations of withdrawal.

The IPN is composed of at least two major subpopulations of GABAergic neurons expressing the α5 nAChR subunit. These populations were identified and classified by their differential expression of the cell-surface adhesion proteins Epyc and Amigo1 (Ables et al., 2017). Epyc-IPN neurons are primarily local interneurons, while Amigo1-IPN neurons project to the Raphe nuclei and the laterodorsal tegmentum. Moreover, silencing of Amigo1-IPN neurons using membrane-tethered toxins prevents conditioned place preference for nicotine, whereas silencing Epyc-cells has no effect on that phenotype (Ables et al., 2017), suggesting that they may serve different functions in the circuit responsible for behavioral changes to nicotine. To investigate this, we used a chemogenetic approach to determine the role of these IPN subpopulations in driving behaviors associated with nicotine withdrawal in mice. Our results reveal separable contributions of the Amigo1 population to the somatic and affective phenotypes of withdrawal.

## 2. Materials and methods

### 2.1. Animals

All experimental procedures were approved by the University of Illinois Chicago Institutional Animal Care Committee and met the NIH guidelines for care and use of laboratory animals. The following transgenic mouse lines were obtained from the Mutant Mouse Research Resource Center (Ables et al., 2017): (Amigo1-cre)NP210Gsat/Mmucd (stock#: 036163-UCD) and (Epyc-cre)KR363Gsat/Mmucd (stock#: 036145-UCD). Mice were genotyped by PCR on genomic tail DNA using primers from the Washington University Genotyping Core Facility. Cre mouse lines were backcrossed at least 10 generations to BL/6 J (Jackson Labs stock no.000664) before behavior testing. Male and female adult mice were used in comparable numbers for all experiments. A total of 213 animals were used in this study. Mice were randomly assigned to experimental groups in numbers comparable to those reported in published literature. Animals were group housed under constant temperature (21–23 °C) and light/dark cycle (14 h/10 h) with access to food and water *ad libitum*.

### 2.2. Stereotaxic delivery of AAV-encoded DREADDs

AAV9-hsyn-DIO-mCherry, AAV9-hsyn-DIO-hM4D(Gi)-mCherry and AAV9-hsyn-DIO-hM3D(Gq)-mCherry were obtained from Addgene (viral prep #50459, #44362, # 44361 respectively; ≥ 1 × 10^13^ vg/mL titer) (Krashes et al., 2011) and kept at −80 °C prior to use. On the day of surgery, the virus was thawed and diluted 1:3 in ddH_2_0. Mice were anesthetized using >2 % isoflurane in air (Somnosuite, Kent Scientific) before mounting into a stereotaxic frame (Stoelting). Isoflurane (1.5–3.5 %) was maintained throughout the procedure and body temperature kept at 37 °C using a heating pad. After exposing the skull, a burr hole was drilled to enable the injection of solutions containing AAV into the IPN (in mm, relative to bregma: −3.400 A/P, 0.666 M/L and 4.854 D/V, at a lateral angle of 8 °) using a 35G needle mounted to a 10 μL Nanofil syringe and driven by a UMPI perfusion pump (WPI). After 500 nL of AAV solution was administered at 250 nL/min followed by a 5-min rest, the needle was withdrawn at a rate of 1 mm/min prior to closing the incision with wound clips. Mice were then allowed to recover before returning to their home cage.

### 2.3. Nicotine exposure

Mice were chronically exposed to nicotine by means of osmotic pumps implanted *via* an incision between the scapulae that delivered a constant subcutaneous flow of 0.5 μL/h for 14 days (RWD Bioscience). Pumps were filled with 0.9 % saline (SAL) for controls, or saline supplemented with nicotine hydrogen tartrate (NIC) (with no adjustment to pH) at a concentration adjusted to individual body weight such that each mouse received nicotine at 1.0 mg/kg/h, expressed as free-base weight. (−)-Nicotine hydrogen tartrate salt was from Sigma-Aldrich. Osmotic pump implantation surgeries were performed a minimum of two weeks after stereotaxic AAV injections.

### 2.4. Behavioral procedures

Behavior experiments were performed on the 14th day following osmotic pump implantation and at least 4 weeks following AAV injection. Each experimental group included animals from at least two different mice litters, surgery days and behavioral testing days such that 2–7 independent replicates per group are shown. Animals were between 20 and 32 weeks of age on the day of testing. Animals were acclimatized to the experimenter, the behavior room and to i.p. injections (using 0.9 % saline) for 2 days before testing. Experiments were conducted in the light phase of the cycle, between 2 and 7 h after the lights-on time. All mice were injected i.p. with 1 mg/kg Clozapine-N-Oxide (CNO) and 3 mg/kg of mecamylamine (Tocris) dissolved in 0.9 % saline, 30 min and 5 min before the start of testing, respectively. An independent cohort of mice was injected only with saline/CNO to confirm that CNO injection does not alter the behaviors of interest (Supplementary Fig. 1). Another independent cohort of nicotine-exposed mice was injected with saline only as a control for the withdrawal precipitation method.

Testing began with an elevated plus maze (EPM; Stoelting) to assess anxiety-like behavior. Mice were placed on the central platform facing an open arm and allowed to explore for 5 min while being recorded by an overhead camera. Time spent and distance travelled in each arm were obtained using EthoVision XT tracking software (Noldus). Open arm time and distance indices were calculated as time or distance spent in open arms over the sum of open and closed arms (not including the center zone, whose occupancy time is excluded in calculating indices). Animals that remained in the center zone of the maze and didn’t explore open or closed arms were excluded from the analysis (*n* = 1 across entire study, not included in total count).

Immediately after the EPM test, mice were placed in a custom-built square arena (30 cm × 30 cm × 30 cm acrylic box with two opaque and two transparent sides) to examine physical signs of nicotine withdrawal for 15 min. Behavior was video-recorded synchronously by two HD cameras set at 90° angles, 15–30 cm from each transparent side, as well as a third, overhead camera. Videos were scored offline from synchronized videos viewed simultaneously by a blinded technician, using a custom-made push-button device for simultaneous recording of onset, offset and duration of events as square waves collected by a MiniDigi 1B digitizer with Axoscope and analyzed in Clampfit10 (Axon Instrument). Signs were divided into the following categories: 1) grooming-related events including face or body scratching, genital licking or any part of the stereotyped sequential movements of grooming and 2) events indicating physical discomfort including head shaking, body twitching or jumping. In addition, overhead videos were analyzed using EthoVision XT to determine distance travelled, and track plots of their exploration of the arena were generated using ezTrack (Pennington et al., 2019). Illumination for both behavior apparatus was approximately 200 lx, while the closed arms of the EPM led to lower brightness (~80 lx), as measured by a handheld light meter.

### 2.5. Histology

Animals were euthanized approximately 90 min after the end of behavioral testing. Mice were perfused with PBS followed by 4 % paraformaldehyde under deep isoflurane anesthesia and the brains were removed and post-fixed in 4 % paraformaldehyde for 3-4 h and cryo-protected in 30 % sucrose. Coronal 20 μm sections were cut using a cryostat mounted onto charged slides, coverslipped using VectaShield mounting media with DAPI (Vector Laboratories) and visualized with a fluorescence microscope (Keyence BZ-X800). Only animals in which we could confirm virus expression in the IPN were included in the study (excluded animals = 2; not included in the total animal count).

### 2.6. Immunofluorescent labeling

For immunofluorescent labeling, 20 μm coronal sections from fixed brains were transferred “free-floating” into a 24 well plate containing PBS. Slices were permeabilized for 10 min in 0.5 % PBST (PBS + 0.5 % Triton X-100, all chemicals from Sigma-Aldrich, St Louis, MO, unless otherwise noted). After one additional wash with 0.1 % PBST, antigen retrieval was performed by exposing slices to 10 mM sodium citrate in PBS (pH = 6.02) at ~80–85 °C for 30 min. After a 5-min PBS wash, slices were then blocked with 2 % normal goat serum (Gibco, Auckland, NZ) in a buffer containing 50 mM glycine, 0.05 % Tween 20, 0.1 % Triton X-100, and 0.01 % bovine serum albumin in PBS for 1 h. Slices were then exposed to rabbit anti-Glutamic Acid Decarboxylase 65/67 (GAD65/67) antibody (1:250, Sigma-Aldrich G1563) in blocking buffer at 4 °C O/N. 1° Abs were omitted in negative control sections. Following two 5-min washes with 0.1 % PBST, slices were incubated with an Alexa Fluor 488-tagged goat anti-rabbit IgG 2° Ab (1:250, Jackson IR, West Grove, PA) in blocking buffer for 1 h at R/T. After two 5-min washes with 0.1 % PBST and one with PBS, slices were mounted on charged slides with ProLong Gold antifade reagent with DAPI (Cell Signaling Technology, Inc., Danvers, MA). Sections were visualized using epifluorescence microscopy (Keyence B*Z*-X800) with a 60× oil immersion, 1.40 N/A PlanApo objective (Nikon Instruments), and images were post-processed off-line using haze reduction and Z-projection functions for clarity.

### 2.7. Ex-vivo electrophysiology

Independent cohorts of mice were used to assess the functional expression of DREADD. Animals were perfused with ice-cold NMDG-based cutting solution (pH: 7.3–7.4; 300–310 mOsm/L) (Ting et al., 2014) bubbled with 5 % carbogen gas (5 % CO_2_, 95 % O_2_), containing (in mM): 93 NMDG, 2.5 KCl, 1.2 NaH_2_PO_4_, 20 HEPES, 25 glucose, 5 Na-ascorbate, 3 Na-pyruvate, 10 MgSO_4_, 0.5 CaCl_2_. Brains were immediately removed to ice-cold, oxygenated NMDG cutting solution and coronal slices of 350 μm sections containing the IPN were obtained using a VT 1000S vibratome (Leica Microsystems). Slices were then placed in a holding chamber in NMDG-based cutting solution at 35 °C for 15 min, transferred to a chamber containing oxygenated HEPES-supplemented aCSF solution (in mM): 92 NaCl, 2.5 KCl, 1.2 NaH_2_PO_4_, 30 NaHCO_3_, 20 HEPES, 25 glucose, 5 Na-ascorbate, 3 Na-pyruvate, 2 MgSO_4_, 2 CaCl_2_ (pH: 7.3–7.4; 300–310 mOsm/L with mannitol), initially held at 35 °C and allowed to cool to room temperature. Slices were held at room temperature for at least 45 min before recording.

Slices were continuously perfused with oxygenated aCSF throughout recordings. Recording aCSF contained (in mM): 127 NaCl, 1.8 KCl, 26 NaHCO_3_, 12 KH_2_PO_4_, 1.3 MgSO_4_, 2.4 CaCl_2_, 15 glucose (pH: 7.3–7.4; 304–306 mOsm/L). Pipette internal solution contained (in mM): 129.5 K-Gluconate, 6.5 KCl, 2 MgCl_2_, 4 Na-ATP, 0.4 Na-GTP, 0.2 EGTA, 10 HEPES (pH: 7.2; 295 mOsm/L). Recording pipettes were pulled from filamented 1.5 OD, 0.86 ID borosilicate glass (Sutter Instrument) using a Sutter P97 puller and polished to a tip resistance of 4–8 MΩ using a Narishige microforge. Following establishment of a GΩ seal, whole cell mode was established using gentle suction, and cells were held for 2–5 min following patch rupture prior to recording. Solution exchange used a Picospritzer3 (Parker-Hanifin) to drive outflow from a 200 μm applicator held immediately above the neuron, and neurons were allowed to equilibrate to each solution for at least 20 s.

All recordings were done in Clampex10 using a MultiClamp 700B amplifier and a Digidata 1550 digitizer (Axon Instruments). Neurons were visualized with a CCD camera (QImaging) driven by μManager software. Electrophysiology data were analyzed using Clampfit10 and only cells with access resistance <35 mΩ were included. Changes in resting membrane potentials were measured in current clamp mode at 0 pA DC current injection. To quantify membrane excitability, DC current was injected to current clamped neurons to reach a rheobase value, which was defined as firing at a frequency of <0.1 Hz across >20 s; the voltages were compared before and after application of 2 μM CNO.

### 2.8. Statistical analysis

Data were summarized as mean ± SEM and differences among experimental conditions were considered statistically significant at *p* < 0.05. Male and female data were grouped since no main effect of sex or sex interactions were found (statistical details and segregated plots per sex are provided in the supplementary materials). Unpaired, two-tailed *t*-tests were used for comparisons between two groups. For comparisons between three and four groups, one and two-way ANOVAs were used respectively (except when SDs were significantly different between groups in which case the Brown-Forsythe ANOVA was employed). Group comparisons were made using Dunnett multiple comparisons test (or Dunnett’s T3 multiple comparisons test for the Brown-Forsythe ANOVA) and Fisher’s LSD test respectively. *P*-values are represented as: *p < 0.05, **p < 0.01, ***p < 0.005 in all figures. Statistical analyses were performed with GraphPad Prism10.

## 3. Results

**Fig. 1.**
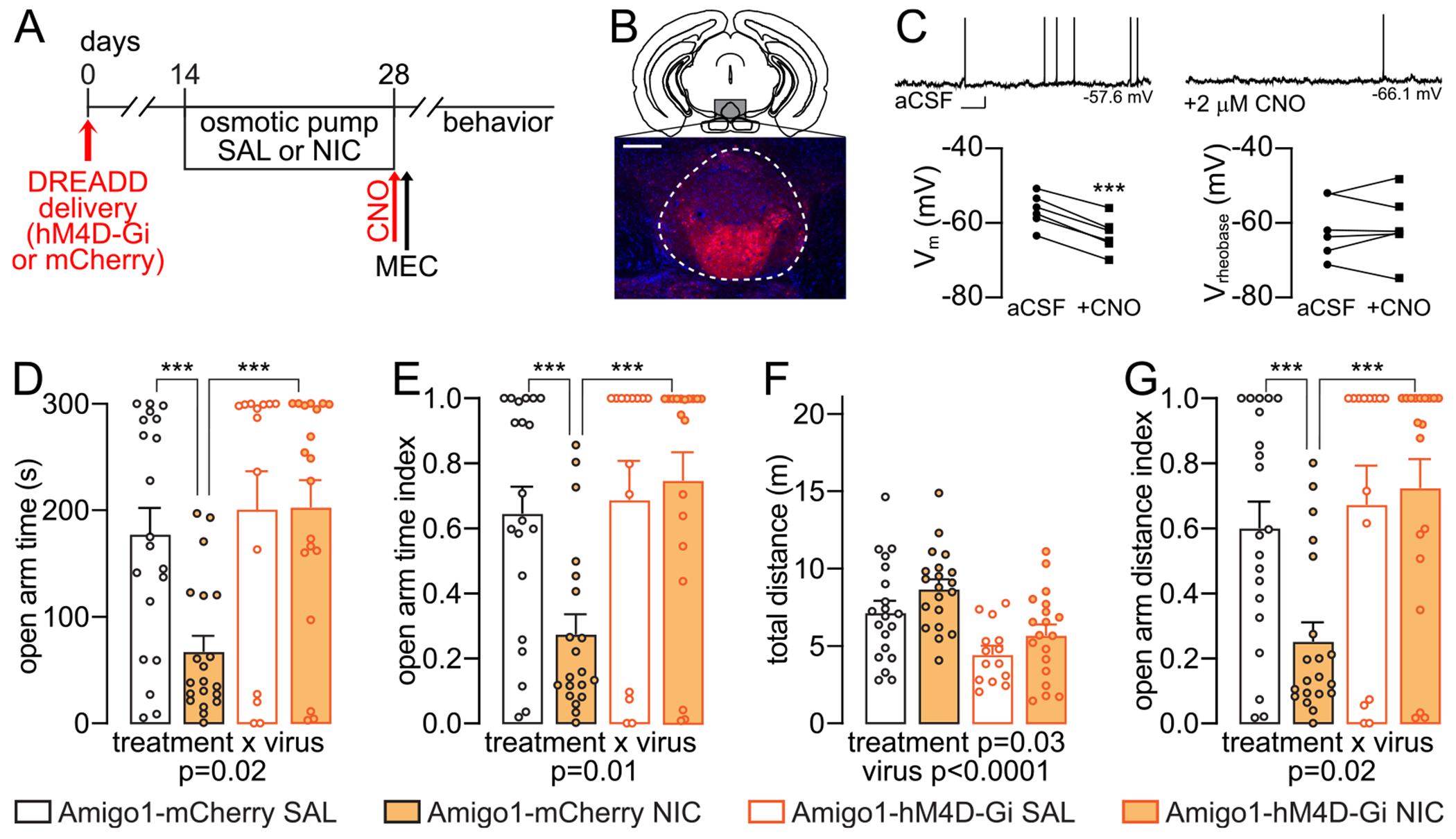
Inhibition of Amigo1-IPN neurons reduces anxiety-like behaviors in mice undergoing nicotine withdrawal. A) Timeline of the experimental design. Experimental groups are as follows: Amigo1-mCherry SAL (*N* = 10 M + 10F), Amigo1-mCherry NIC (*N* = 8 M + 12F), Amigo1 hM4D-Gi SAL (*N* = 9 M + 5F), Amigo1-hM4D-Gi NIC (N = 9 M + 10F). B) Schematic diagram of virus injection target and representative image of virus expression in the IPN (outlined) of Amigo1-cre male and female adult mice. Scale bar: 250 μm. C) *Ex-vivo* electrophysiology. *Top*: Sample traces of action potentials from Amigo1-cre cells expressing hM4D(Gi) in response to aCSF alone or supplemented with 2 μM CNO, with resting membrane potentials as indicated. Scale bar indicates 10 mV and 500 ms and applies to all traces. *Bottom*: CNO application led to a negative shift in resting membrane voltage compared with aCSF alone (*left*) but no apparent change to voltage rheobase (*right*) (*n* = 6, *t*-test). D-G) Elevated plus maze. Maze exploration was assessed by evaluating time spent in the open arms (D: treatment x virus F_(1,69)_ = 5.35) and the total distance travelled in the maze (F: treatment x virus F_(1,69)_ = 0.05, *p* = 0.82; treatment F_(1,69)_ = 4.63; virus F_(1,69)_ = 19.76) as a measure of locomotor activity. In addition, an open arm index was calculated for time spent (E: treatment x virus F_(1,69)_ = 6.69) and distance travelled (G: treatment x virus F_(1,69)_ = 5.79) (full details in Methods). Two-way ANOVA. *p < 0.05, **p < 0.01, ***p < 0.005. There was no main effect of sex, sex x virus, sex x treatment, or sex x virus x treatment interactions (Supplementary Fig. 11 & Table 1).

We first examined the contribution of Amigo1-IPN neurons to behaviors associated with nicotine withdrawal. Amigo1-cre mice (Ables et al., 2017) were infected in the IPN with hM4D-Gi, an inhibitory Designer Receptor Exclusively Activated by Designer Drugs (DREADD) by stereotaxic injection of AAV9-hSyn-DIO-hM4D(Gi)-mCherry (Krashes et al., 2011) (see timeline in Fig. 1A), an adeno-associated virus (AAV) that encodes cre-dependent hM4D-Gi fused with an mCherry fluorophore. Control animals were injected with AAV9-hSyn-DIO-mCherry lacking hM4D-Gi. Viral expression in the IPN was confirmed in all mice *post-mortem* by fluorescence microscopy of coronal cryosections (Fig. 1B), and resembled the distribution across IPN subdivisions of cre-dependent fluorescent marker in Amigo1 animals observed by Ables et al. (Ables et al., 2017) (Supplementary Fig. 2 A–B). Additional cryo-sections were co-labeled with an antibody against GAD65/67, a marker of GABAergic neurons, to confirm that these represent a primarily GABAergic population (Supplementary Fig. 3A). Patch clamp electrophysiology from IPN neurons in acute *ex vivo* brain slices was used to assess the functional impact of the DREADD expression. We quantified resting potential (−56.5 ± 1.8 mV, Fig. 1C *left*) and voltage at the “rheobase” of DC current injection (−61.3 ± 3.2 mV, Fig. 1C *right*) as measures of passive and active membrane properties, respectively. We then applied CNO and quantified the result changes to the resting potential (−63.3 ± 1.9 mV) and voltage at rheobase (−61.0 ± 3.7 mV). We observed a significant hyperpolarization of the resting membrane potential in the presence of CNO (*p* < 0.0001, Fig. 1C *left*), but no apparent change to voltage at rheobase (*p* = 0.87, Fig. 1C *right*). Next, we examined changes to affective behavior in response to nicotine delivered by subcutaneous osmotic pump and precipitated withdrawal, using an elevated plus maze (EPM). Open arm avoidance and increased occupancy of the closed arms were interpreted as reflecting heightened anxiety and were observed in mice chronically exposed to nicotine and injected with mecamylamine to precipitate withdrawal, but not in chronic saline-exposed controls injected with mecamylamine (Fig. 1D–E), nor in mice exposed to nicotine but without precipitation of withdrawal by mecamylamine (Supplementary Fig. 4). We found that inhibition of Amigo1-IPN neurons prior to precipitation of withdrawal reduced anxiety-like behaviors in animals chronically exposed to nicotine as evidenced by an increased open arm exploration compared to their mCherry counterparts (p < 0.0001, Fig. 1D; p < 0.0001, Fig. 1E) which also received i.p. CNO injection. This reduction of anxiety-like behavior to the levels of nicotine-naïve animals was not explained by changes in locomotor activity (Fig. 1F), since the distance travelled in the open arms was larger than that in the closed arm, resulting in a significantly higher open arm distance index than nicotine controls (*p* ≤0.0001, Fig. 1G). In contrast, inhibition of Amigo1-IPN neurons in nicotine naïve animals did not change the time spent (*p* = 0.51, Fig. 1D; *p* = 0.73, Fig. 1E) or distance travelled (*p* = 0.56, Fig. 1G) in the open arms of the maze compared to their mCherry counterparts. Thus, the described effect that inhibition of the Amigo1 subpopulation of IPN neurons has on EPM exploration is specific to nicotine-exposed mice.

**Fig. 2.**
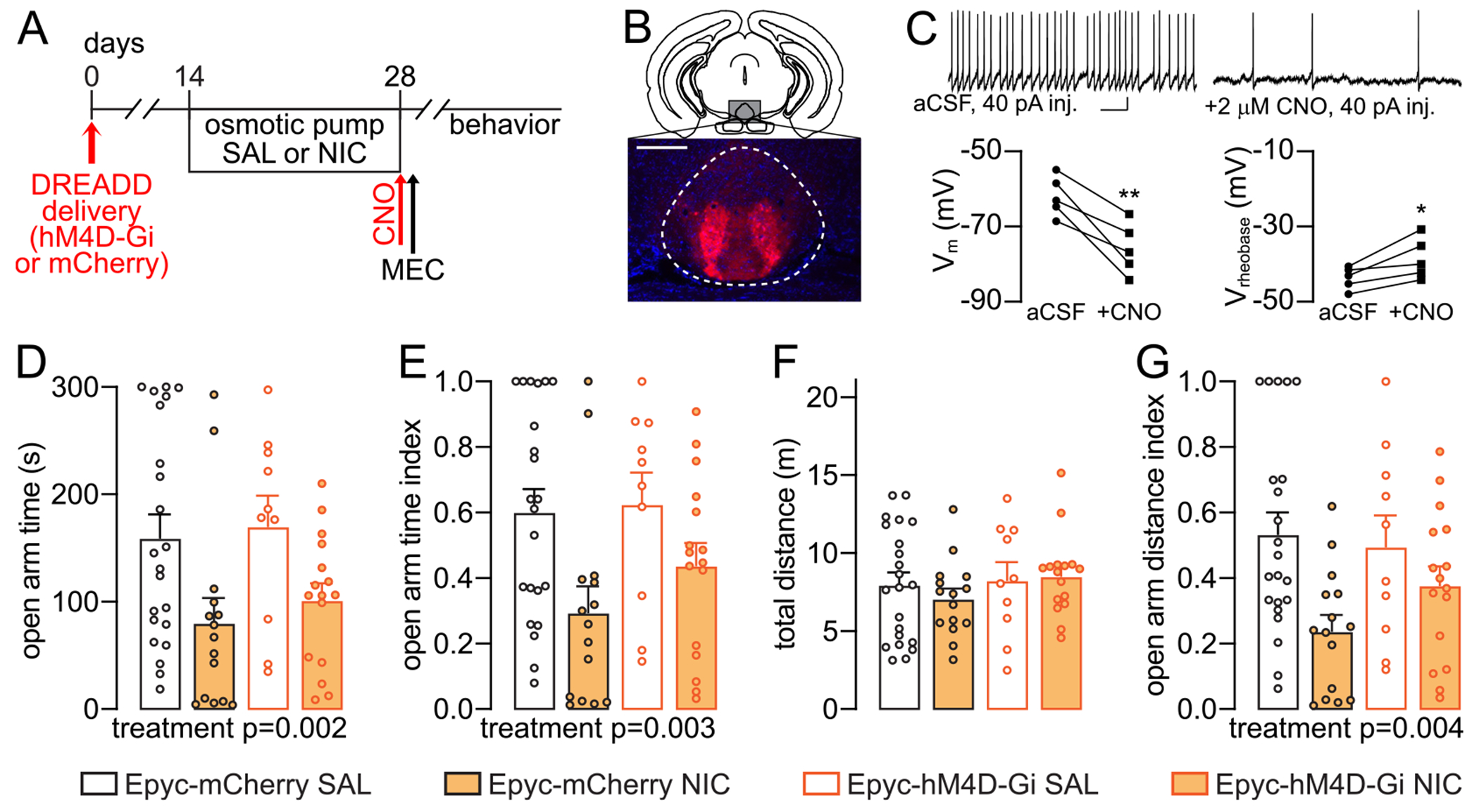
Inhibition of Epyc-IPN neurons fails to alter anxiety-like behaviors in nicotine exposed mice after precipitated withdrawal. A) Timeline of the experimental design. Experimental groups are as follows: Epyc-mCherry SAL (*N* = 12 M + 10F), Epyc-mCherry NIC (*N* = *7* M + 8F) and Epyc-hM4D-Gi SAL (*N* = 4 M + 6F), Epyc-hM4D-Gi NIC (N = 8 M + 8F). B) Schematic diagram of virus injection target and representative image of virus expression in the IPN (outlined) of Epyc-cre male and female adult mice. Scale bar 250 μm. C) *Ex-vivo* electrophysiology. *Top*: Sample traces of action potentials from Epyc-cre cells expressing hM4D-Gi in response to aCSF alone or supplemented with 2 μM CNO. To reveal an effect on action potential activity, a DC current injection of 40 pA was made to stimulate repetitive firing. Scale bar indicates 10 mV and 500 ms and applies to all traces. *Bottom*: CNO application led to a negative shift in resting membrane voltage compared with aCSF alone (*left*) (*n* = 5, t-test). A positive shift in the rheobase for action potential voltage (f < 0.1 Hz) (*right*) was observed following CNO application (n = 5, t-test). D-G) Elevated plus maze. Maze exploration was assessed by evaluating time spent in the open arms (D: treatment x virus F_(1,59)_ = 0.05, p = 0.82, treatment F_(1,59)_ = 10.48; virus F_(1,59)_ = 0.49, *p* = 0.48) and the total distance travelled in the maze (F: treatment x virus F_(1,59)_ = 0.50, p = 0.48; treatment F_(1,59)_ = 0.14, *p* = 0.70; virus F_(1,59)_ = 1.12, *p* = 0.29) as a measure of locomotor activity. In addition, an open arm index was calculated for time spent (E: treatment x virus F_(1,59)_ = 0.56, *p* = 0.46, treatment F_(1,59)_ = 9.71; virus F_(1,59)_ = 1.11, *p* = 0.30) and distance travelled (G: treatment x virus F_(1,59)_ = 1.68, *p* = 0.20, treatment F_(1,59)_ = 9.14; virus F_(1,59)_ = 0.57, *p* = 0.45) (full details in Methods). Two-way ANOVA. *p < 0.05, **p < 0.01, ***p < 0.005. There was no main effect of sex, sex x virus, sex x treatment, or sex x virus x treatment interactions (Supplementary Fig. 12 & Table 2), except for the total distance travelled that a sex x virus interaction was observed (Supplementary Fig. 12 & Table 2).

We then examined the contribution of a second population of neurons, marked by the gene *Epyc,* to affective behaviors (Fig. 2A). These neurons exhibited a distinct expression pattern of DIO-mCherry compared with Amigo1 neurons (Supplementary Fig. 2C–D) (Ables et al., 2017), and also showed co-labeling with a GAD65/67 antibody marking GABAergic neurons (Supplementary Fig. 3B). The functional impact of hM4D-Gi DREADD expression in this subpopulation was also assessed using *ex vivo* brain slice electrophysiology (Fig. 2B–C). Epyc neurons didn’t fire spontaneously at rest, so a small 40 pA current was injected to illustrate the effect of CNO on action potential firing (Fig. 2C, *top*). Application of CNO resulted in a significant hyperpolarization of the resting membrane potential (from −61.7 ± 2.4 mV to −75.7 ± 3.1 mV, *p* = 0.0072, Fig. 2C *left*), and an increase of the voltage at rheobase (from −43.5 ± −1.3 mV to −38.3 ± 2.4 mV, *p* = 0.03, Fig. 2C *right*). Next, we evaluated affective behaviors of these mice in the EPM test (Fig. 2D–G). Nicotine-exposed animals displayed a typical pattern of enhanced anxiety evidenced by the reduced time spent (Fig. 2D–E) and open arm distance travelled (Fig. 2G) compared to saline exposed controls. However, inhibition of Epyc-IPN neurons did not alter the behavior of nicotine-exposed or nicotine naïve mice (Fig. 2D,E,G) nor the total distance travelled in the maze (Fig. 2F) after mecamylamine injection. Taken together with the results shown in Fig. 1, it is conceivable that enhanced activity of Amigo1-, but not Epyc-IPN neurons is required for sustaining anxiety-like behaviors in nicotine-exposed mice after precipitated withdrawal.

**Fig. 3.**
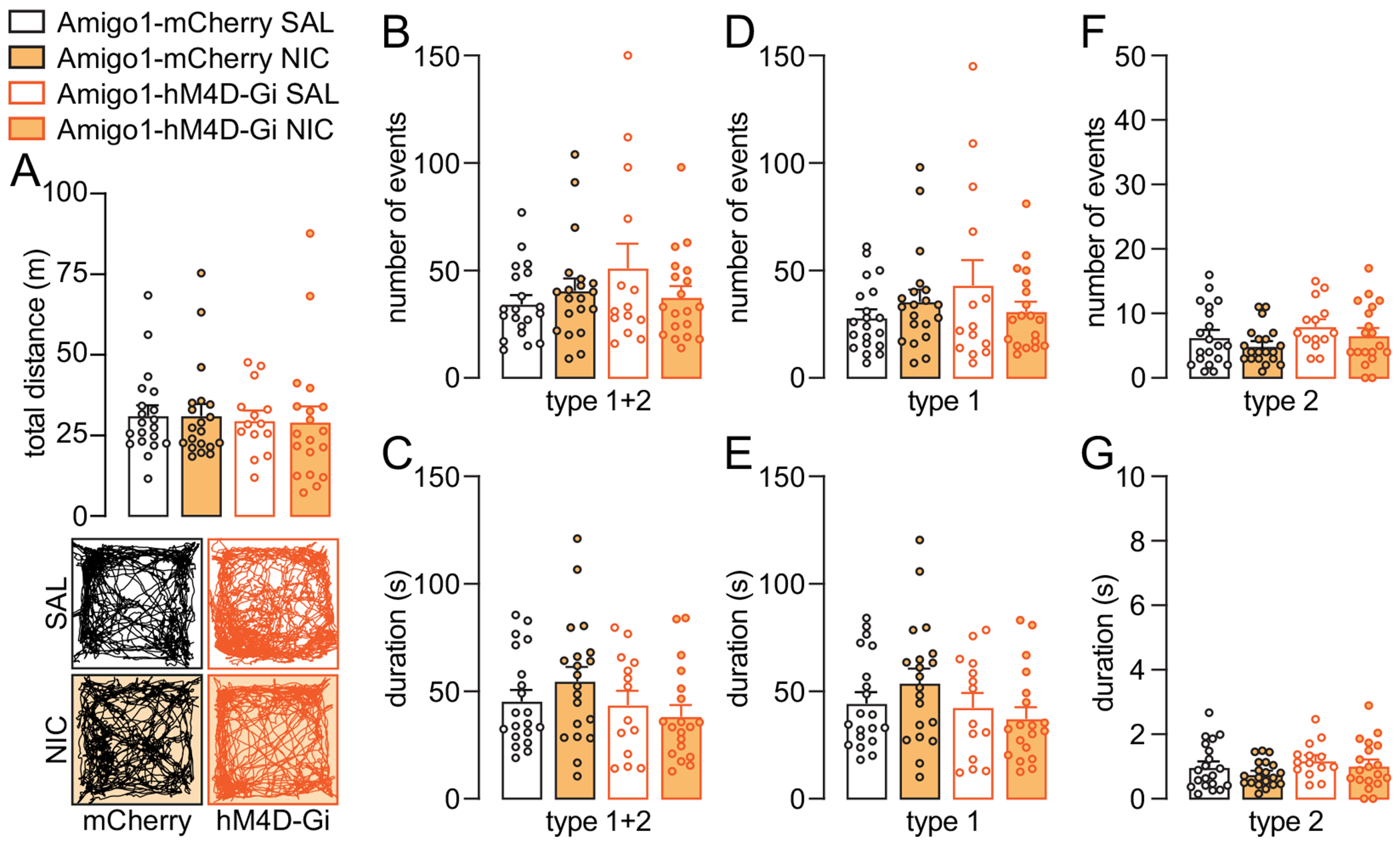
Precipitated withdrawal after nicotine exposure does not significantly alter expression of somatic behaviors, nor does inhibition of Amigo1-IPN neurons. A) *Top*: Total distance travelled in the open arena was similar for all experimental groups (treatment x virus F_(1,69)_ = 0.005, *p* = 0.95; treatment F_(1,69)_ = 0.004, p = 0.95; virus F_(1,69)_ = 0.23, *p* = 0.63). *Bottom*: example track plot for each group. B-G) Somatic signs. Behaviors were grouped in two types: 1) grooming-related (D-E) and 2) shakes & twitches (F-G) (full description in Methods). No behavioral change was apparent between groups with respect to the total number (B: treatment x virus F_(1,69)_ = 2.63, *p* = 0.11; treatment F_(1,69)_ = 0.36, *p* = 0.55; virus F_(1,69)_ = 1.26, *p* = 0.27) or duration of these somatic behaviors (C: treatment x virus F_(1,69)_ = 1.64, p = 0.20; treatment F_(1,69)_ = 0.12, *p* = 0.73; virus F_(1,69)_ = 2.55, *p* = 0.12). Similarly, no changes in number (D: treatment x virus F_(1,69)_ = 2.69, p = 0.11; treatment F_(1,69)_ = 0.15, p = 0.70; virus F_(1,69)_ = 0.75, *p* = 0.40) or duration (E: treatment x virus F_(1,69)_ = 1.66, p = 0.20; treatment F_(1,69)_ = 0.14, *p* = 0.71; virus F_(1,69)_ = 2.69, p = 0.11) of type 1 or type 2 events (F: treatment x virus F_(1,69)_ = 0.0004, *p* = 0.98; treatment F_(1,69)_ = 2.00, *p* = 0.16; virus F_(1,69)_ = 2.92, *p* = 0.09; G: treatment x virus F_(1,69)_ = 0.04, *p* = 0.84; treatment F_(1,69)_ = 1.66, p = 0.20; virus F_(1,69)_ = 2.52, p = 0.12) were evidenced. Two-way ANOVA. There was no effect of sex x virus x treatment interactions (Supplementary Fig. 13 & Table 3), except for the number of type 1 events where sex x virus x treatment interaction was observed, which was driven by differences between male and female mice of the Amigo1-Gi SAL group (Supplementary Fig. 13 & Table 3A–D).

**Fig. 4.**
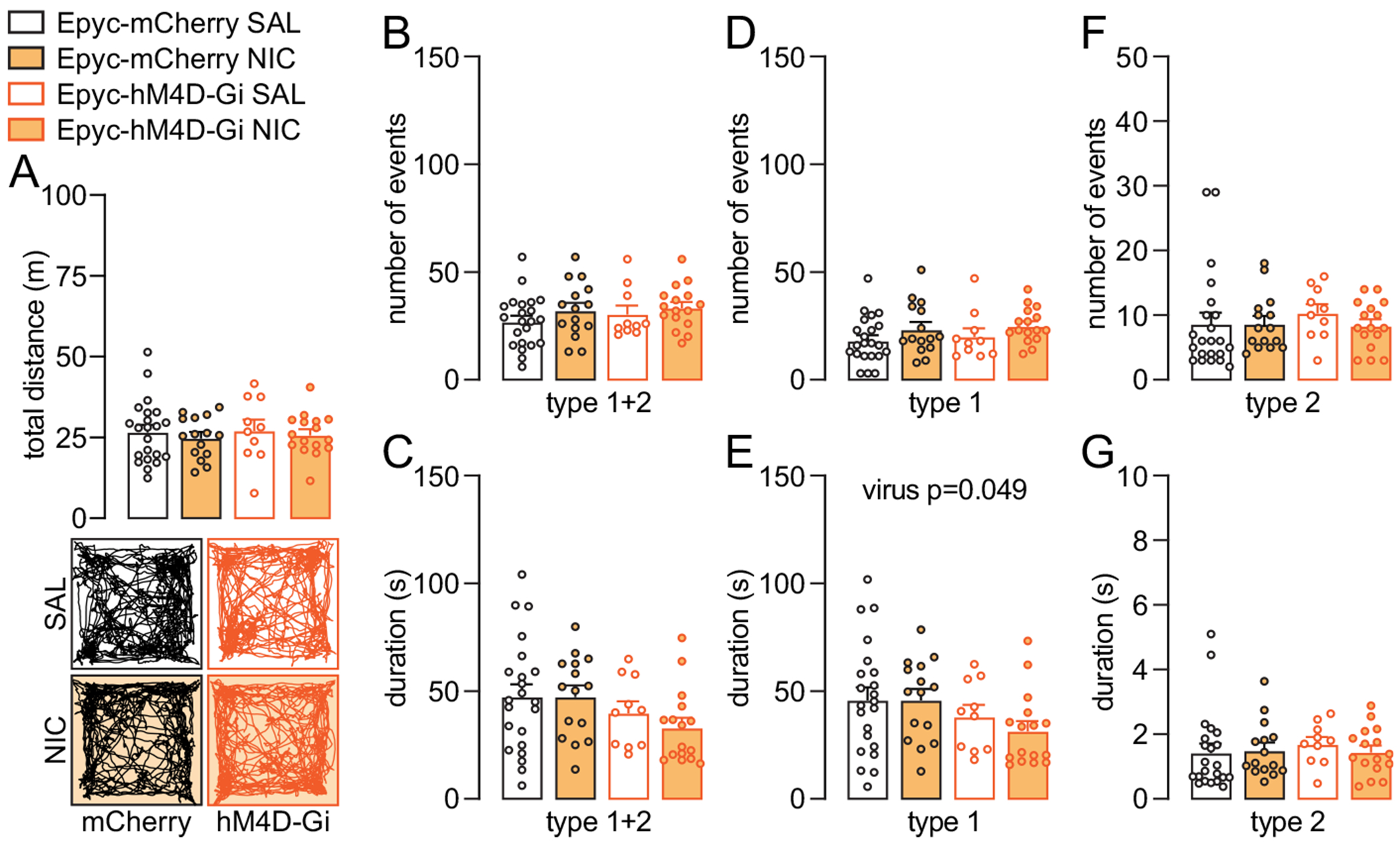
Precipitated withdrawal after nicotine exposure does not change somatic behaviors, nor does inhibition of Epyc-IPN neurons. A) *Top*: Total distance travelled in the arena was similar for all experimental groups (treatment x virus F_(1,59)_ = 0.01, *p* = 0.92; treatment F_(1,59)_ = 0.53, *p* = 0.47; virus F_(1,59)_ = 0.10, *p* = 0.75). *Bottom*: example track plot for each group. B-G) Somatic signs. Behaviors were grouped in two types: 1) grooming-related (D-E) and 2) shakes & twitches (F-G) (full description in Methods). No behavioral change was apparent between groups with respect to the total number (B: treatment x virus F_(1,59)_ = 0.14, p = 0.71; treatment F_(1,59)_ = 1.76, *p* = 0.19; virus F_(1,59)_ = 0.60, *p* = 0.44) or duration of these somatic behaviors (C: treatment x virus F_(1,59)_ = 0.39, *p* = 0.53; treatment F_(1,59)_ = 0.38, *p* = 0.54; virus F_(1,59)_ = 3.91, *p* = 0.05). No changes in the number of type 1 events were evidenced (D: treatment x virus F_(1,59)_ = 0.004, p = 0.95; treatment F_(1,59)_ = 3.35, *p* = 0.07; virus F_(1,59)_ = 0.38, p = 0.54) and a small reduction of the duration of type 1 events was observed between hM4D-Gi and mCherry groups.(E: treatment x virus F_(1,59)_ = 0.36, p = 0.55; treatment F_(1,59)_ = 0.36, p = 0.55; virus F_(1,59)_ = 4.04). Type 2 events were similar between all groups both in number (F: treatment x virus F_(1,59)_ = 0.44, *p* = 0.51; treatment F_(1,59)_ = 0.43, p = 0.51; virus F_(1,59)_ = 0.22, *p* = 0.64) and duration (G: treatment x virus F_(1,59)_ = 0.39, p = 0.54; treatment F_(1,59)_ = 0.11, *p* = 0.74; virus *p* = 0.68). Two-way ANOVA. There was no main effect of sex, sex x virus, sex x treatment, or sex x virus x treatment interactions (Supplementary Fig. 14 & Table 4).

In addition to anxiety-like behaviors, alterations in physical signs within the sensorimotor domain such as grooming, scratching, twitching and other stereotyped actions have been described as somatic manifestations of nicotine withdrawal in rodents (Damaj et al., 2003; Malin et al., 1994; Malin et al., 1992). Therefore, immediately after the EPM test we recorded open field behavior for 15 min and measured two types of somatic behaviors: 1) grooming and 2) shakes & twitches (detailed description in Methods). To establish that these animals exhibit stereotypical somatic signs, we first compared a control group of mice implanted with osmotic pumps but given acute saline instead of mecamylamine with the precipitated withdrawal groups. We found that both Amigo1-cre and Epyc-cre animals given mecamylamine exhibited a significant increase in the number of type 1 and type 2 somatic signs respectively, compared to the saline injected controls (Supplementary Fig. 5). However, when nicotine-exposed animals receiving mecamylamine were compared to saline-exposed mecamylamine injected animals, neither the number of (Fig. 3B,D,F) nor the duration of these behaviors (Fig. 3C,E,G) was affected by nicotine exposure compared to saline mCherry controls. Moreover, inhibition of Amigo1-IPN neurons did not alter any of these behaviors in nicotine exposed and nicotine naïve animals when comparing CNO-injected animals expressing hM4D-Gi or mCherry in their IPN (Fig. 3B–G). No change was observed either in the distance travelled in the maze by all the experimental groups (Fig. 3A). Similar findings were observed for Epyc-cre animals, where nicotine exposure prior to mecamylamine injection and/or inhibition of Epyc-IPN neurons did not change the distance travelled in the arena (Fig. 4A) or any of the somatic behaviors assessed (Fig. 4B–G). These findings were in contrast with a small difference in somatic signs achieved by precipitating withdrawal in the nicotine-exposed groups by mecamylamine injection, compared with control saline injections (Supplementary Fig. 5). We also tested whether changes in somatic signs might occur earlier after mecamylamine injection (*i.e.* over the period during which changes in affective behavior were observed in our paradigm) or might be quantitatively different if collected without prior EPM testing, but no difference was observed (Supplementary Fig. 6). Moreover, reducing the activity of Epyc or Amigo1-IPN neurons was insufficient to alter these behaviors in both nicotine exposed and nicotine naïve mice (Figs. 3, 4).

**Fig. 5.**
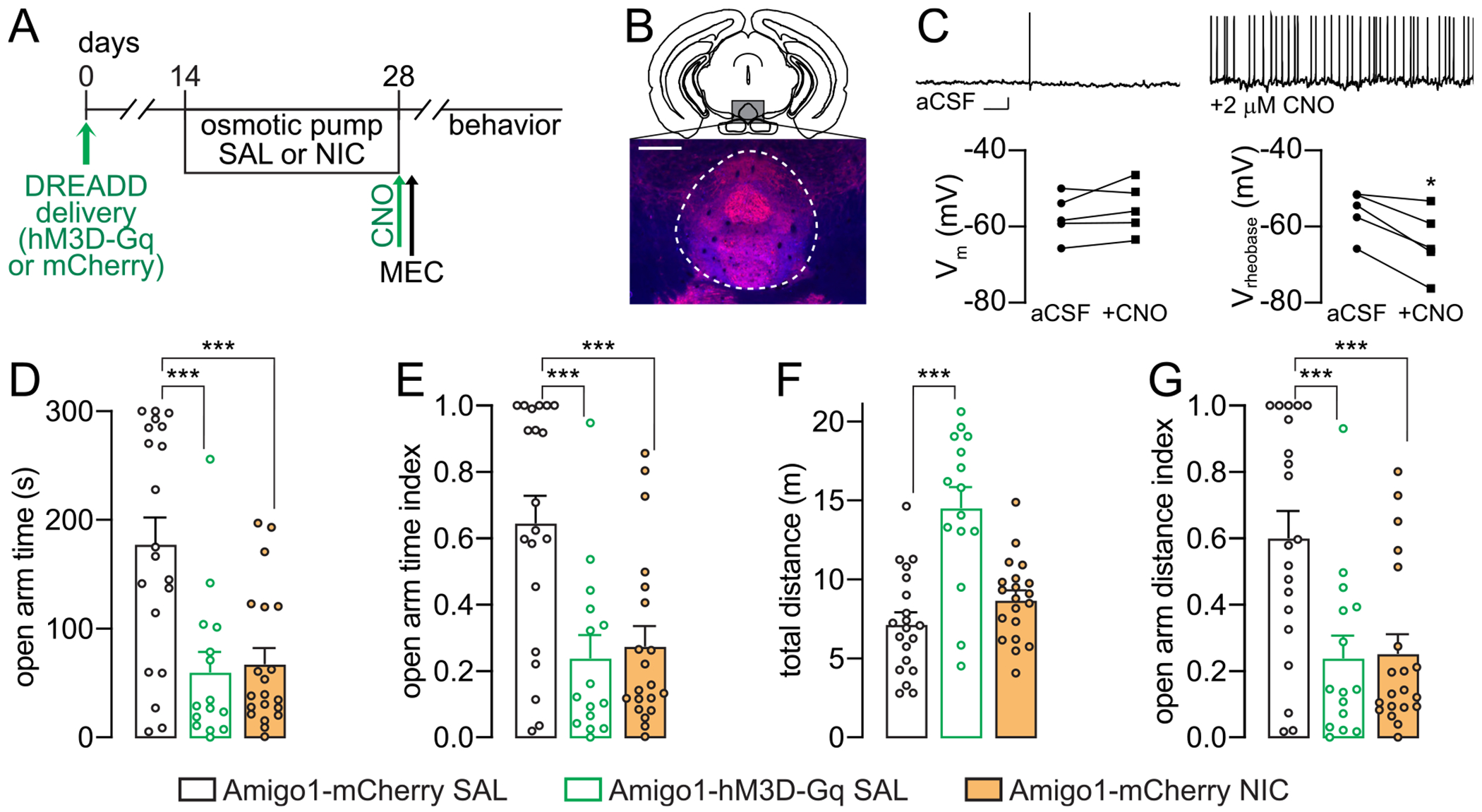
Stimulation of Amigo1-IPN neurons is sufficient to elicit anxiety-like behaviors in nicotine-naïve mice. A) Timeline of the experimental design. Experimental groups are as follows: Amigo1-mCherry SAL (N = 10 M + 10F), Amigo1-hM3D-Gq SAL (N = 9 M + 6F), Amigo1-mCherry NIC (N = 8 M + 12F). B) Schematic diagram of virus injection target and representative image of virus expression in the IPN (outlined) of Amigo1-cre male and female adult mice. Scale bar: 250 μm C) *Ex-vivo* electrophysiology. *Top*: Sample traces of action potentials from Amigo1-cre cells expressing hM3D-Gq in response to aCSF alone or supplemented with 2 μM CNO. Scale bar indicates 10 mV and 500 ms and applies to all traces. *Bottom*: A negative shift in the rheobase for action potential voltage (f < 0.1 Hz) was observed following CNO application (*right*) but no change in resting membrane potential was seen (*left*) (n = 5, t-test). D-G) Elevated plus maze. Maze exploration was assessed by evaluating time spent in the open arms (D: F(_2, 44.10_) = 12.42, *p* < 0.0001) and the total distance travelled in the maze (F: F(_2, 52_) = 19.94, p < 0.0001) as a measure of locomotor activity. In addition, an open arm index was calculated for time spent (E: F(2, 52) = 10.71, *p* = 0.0001) and distance travelled (G: F_(2, 52)_ = 9.39, *p* = 0.0003) (full details in Methods). One-way ANOVA. *p < 0.05, **p < 0.01, ***p < 0.005. There was no main effect of sex or sex x treatment interaction (Supplementary Fig. 15 & Table 5).

To determine the extent to which Amigo1 cells regulate anxiety-like behaviors we next asked whether stimulation of Amigo1-IPN neurons is sufficient to elicit such behaviors in the absence of nicotine exposure (Fig. 5A for timeline). For that purpose, Amigo1-cre animals were infected in their IPN by AAV9-hSyn-DIO-hM3D(Gq)-mCherry, which delivers an excitatory hM3D-Gq cre-dependent DREADD fused with mCherry, or AAV-DIO-mCherry as a control. To test the effect of the DREADD on IPN neuron activity, we recorded hM3D-Gq-mCherry IPN neurons with patch clamp electrophysiology as before (Fig. 5B–C). In the presence of CNO, Amigo1 neurons showed a marked increase in action potential frequency, necessitating a negative DC current injection to quantify voltage at rheobase, which shifted from −56.1 ± 2.6 mV to −64.1 ± 3.9 mV upon CNO exposure (*p* = 0.01, Fig. 5C *right*), indicating that CNO could effectively potentiate excitability in hM3D-Gq expressing Amigo1-cre neurons. Conversely, we recorded no significant change to passive properties measured by resting potential, which was −57.8 ± 2.7 mV before and −55.6 ± 3.0 mV after CNO exposure (*p* = 0.22, Fig. 5C *left*). We then quantified affective behaviors in Amigo1-hM3D-Gq using the EPM. Here, we found that stimulation of Amigo1-IPN neurons was sufficient to reduce the exploration of the open arms in nicotine-naïve mice. This included a reduced time spent in the open arms of the maze (*p* = 0.0008, Fig. 5D) leading to a reduced open arm time index (*p* = 0.0004, Fig. 5E) compared to saline controls. This pattern remained after accounting for increased locomotor activity in these animals (*p* < 0.0001, Fig. 5F) as shown by the open arm distance index (*p* = 0.0012, Fig. 5G). Taken together, these results indicate that stimulating Amigo1-IPN neurons is anxiogenic in nicotine-naïve mice.

**Fig. 6.**
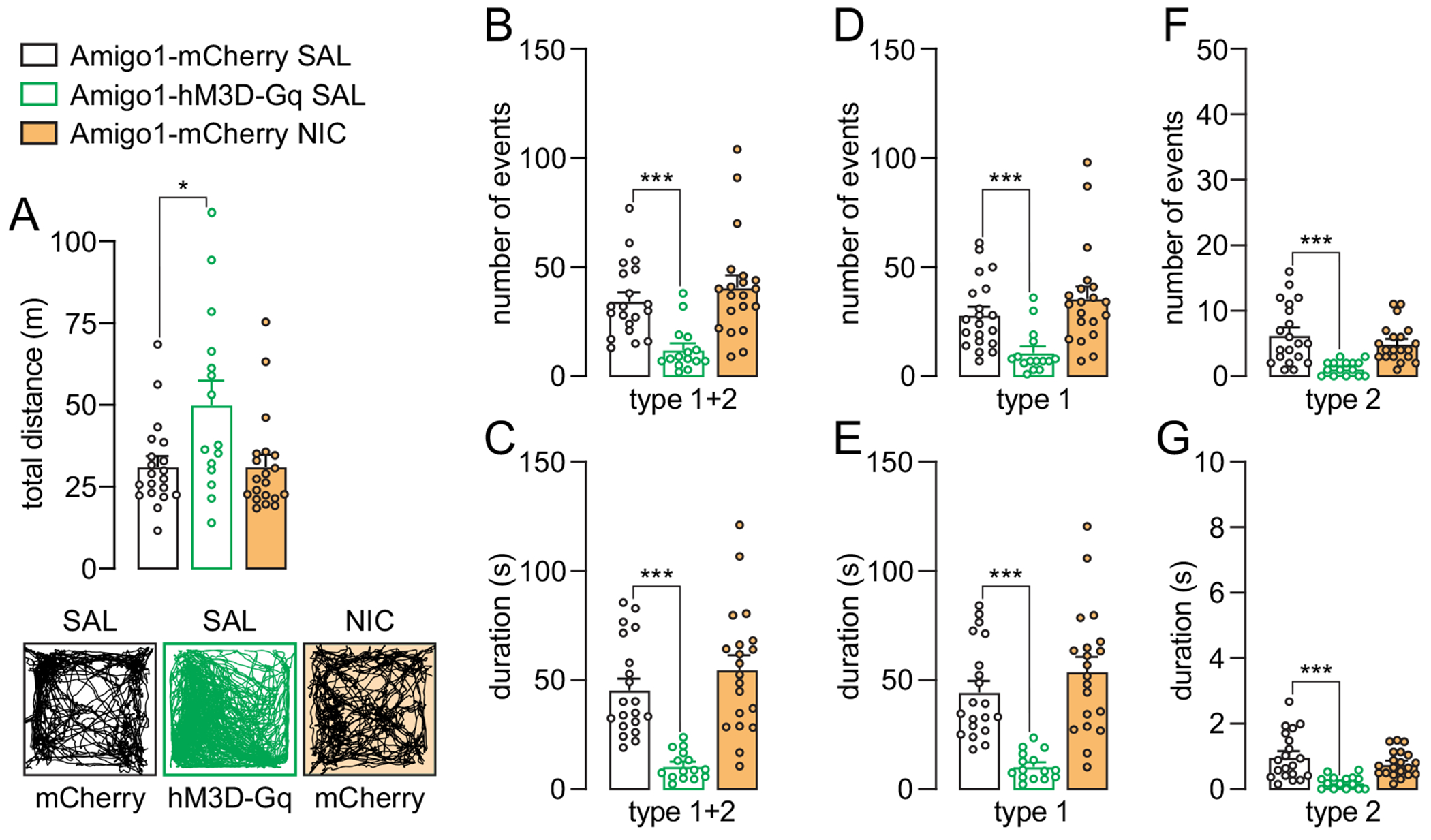
Stimulation of Amigo1-IPN neurons reduces somatic behaviors in nicotine-naïve mice while increasing overall locomotor activity. A) *Top*: Total distance travelled in the arena was significantly higher for the Amigo1-hM3D-Gq group (F_(2, 27.36)_ = 4.81, *p* = 0.02) *Bottom*: example track plot for each group. B-G) Somatic signs. Behaviors were grouped in two types: 1) grooming-related (D, E) and 2) shakes & twitches (F, G) (full description in Methods). The total number of events (B: F_(2, 42.66)_ = 11.90 p < 0.0001) and the duration (C: F_(2, 38.17)_ = 21.41, p < 0.0001) of somatic behaviors was significantly reduced following stimulation of Amigo1-IPN neurons. Similar changes in the number (D: F_(2, 52)_ = 8.54, *p* = 0.0006) and duration (E: F_(2, 38.04)_ = 20.91, p < 0.0001) of type 1 and type 2 events (F: F_(2, 34.92)_ = 11.85, p = 0.0001; G: F_(2, 33.17)_ = 12.07, p = 0.0001) were observed. One-way ANOVA. *p < 0.05, **p < 0.01, ***p < 0.005. There was no main effect of sex or sex x treatment interaction (Supplementary Fig. 16 &Table 6).

Next, we tested whether Amigo1-IPN stimulation would also affect somatic behaviors typically associated with nicotine withdrawal (Fig. 6). In contrast with our observations using inhibitory DREADD, stimulation of Amigo1-IPN neurons significantly reduced the total number (p < 0.0001, Fig. 6B) and duration (p < 0.0001, Fig. 6C) of somatic behaviors. This change was also reflected in the number (p = 0.001, Fig. 6D) and duration (p < 0.0001, Fig. 6E) of type 1 and type 2 behaviors (*p* = 0.0002, Fig. 6F; p = 0.0002, Fig. 6G) compared with nicotine-naïve controls suggesting that Amigo1-IPN neurons are part of the circuit responsible for these behaviors under basal conditions. This manipulation also resulted in an increase in the locomotor activity measured as an increase in total distance travelled in the arena (*p* = 0.048, Fig. 6A), consistent with previous associations between IPN activity and heightened locomotor behavior (Lu et al., 2020). To test the specificity of this effect to the Amigo1-IPN population, we performed a similar experiment to stimulate the Epyc-IPN population, by injecting hM3D-Gq-mCherry into the IPN of Epyc-cre mice and quantifying somatic and affective signs in response to CNO with the same behavioral paradigms. However, we found that activating Epyc-IPN neurons with i. p. CNO did not elicit any changes in the affective (Supplementary Fig. 7) or somatic (Supplementary Fig. 8) behaviors studied.

Taken together, these results indicate that Amigo1-IPN neurons are differentially involved in two behavioral phenotypes associated with withdrawal after nicotine exposure such that their stimulation in the absence of nicotine elicits clear changes in opposite directions (*i.e.* increased anxiety-like behavior and reduced somatic signs).

## 4. Discussion

The IPN plays a crucial role in the somatic and affective behaviors that characterize nicotine withdrawal syndrome (Klenowski et al., 2022; Salas et al., 2009; Zhao-Shea et al., 2013). Several lines of evidence had indicated that IPN is heterogeneous with different cell types marked by different calcium binding proteins, neuropeptides, and nAChRs (Hsu et al., 2013; Shih et al., 2014; Zhao-Shea et al., 2013). However, Ables and colleagues provided the first description of non-overlapping neuronal subpopulations with separate behavioral outcomes (Ables et al., 2017). Here, we leveraged this distinction to independently target Amigo1 or Epyc neuronal subpopulations within the IPN. We found that Amigo1-IPN cells are essential in regulating anxiety-like behavior in mice navigating an EPM. By effectively modulating the activity of this subset of GABAergic IPN neurons we could prevent or induce the characteristic affective phenotype of nicotine withdrawal. Thus, activity within the subpopulation of Amigo1-IPN neurons is both necessary for withdrawal-associated anxiogenesis, and sufficient to induce an anxiogenic phenotype in the EPM in naïve mice. This agrees with the evidence establishing the IPN as a mediator of negative emotional states, such as fear and anxiety both in basal and nicotine-withdrawal conditions (Molas et al., 2017). In contrast, we did not find any detectable behavioral effects following chemogenetic modulation of a separate pool of GABAergic neurons in the IPN, marked by expression of Epyc. Together, our results indicate that different neuronal populations within the IPN drive distinct behavioral phenotypes following chronic nicotine exposure and/or chemogenetic manipulation followed by administration of mecamylamine. Furthermore, we are not aware of any potential cross-talk between CNO and mecamylamine that could account for the behavioral effects observed following chemogenetic inhibition of the Amigo-expressing neuronal population.

In current clamp electrophysiology experiments to validate the effects of CNO application, we also noted that Amigo1 neurons appeared markedly more excitable than Epyc neurons from naïve brains prior to the exposure of the CNO. When passive and active voltage properties from these groups are compared directly, we observed that while the resting voltages for Amigo1 (−58.6 ± 1.6 mV) and Epyc neurons (−57.1 ± 1.5 mV) were not different (*n* ≥ 11, *p* = 0.50, unpaired, two-tailed *t*-test), the “Rheobase voltage” at which naïve Amigo1 neurons begin to exhibit spontaneous firing (−58.9 ± 2.2 mV) was significantly depolarized from that for Epyc neurons (−47.4 ± 1.5 mV), (n ≥ 11, *p* = 0.0002, unpaired, two-tailed t-test). Those results demonstrate that Amigo1 neurons are more excitable than Epyc neurons and confirm that in addition to their genomic and anatomical distinctions (Ables et al., 2017), these populations can also be clearly distinguished by their neurophysiological properties. Given their separable roles in driving the withdrawal phenotype, it will be valuable to explore how the distinct firing behaviors (of Amigo1 neurons, in particular) are causally involved in driving the observed behaviors using modern *in vivo* imaging methods. Moreover, as chronic nicotine or mecamylamine exposure have been found to directly act upon IPN neurons (Arvin et al., 2019; Salas et al., 2009), future studies exploring how and when nicotine cell autonomously alters channel expression and function and synaptic plasticity will aid in elucidating molecular and cellular mechanisms acting at Amigo1-IPN neurons to propagate withdrawal phenotypes.

In contrast to studies linking nicotine and affective behavior, somatic manifestations of nicotine withdrawal are more variable in the behaviors evaluated and how they are assessed. Although many experimenters use a combined withdrawal score, others report that nicotine withdrawal does not elicit an increase in all somatic signs analyzed (Klenowski et al., 2022; Malin et al., 1992; Zhao-Shea et al., 2013). Recognizing the challenges of assessing this aspect of the syndrome, we implemented a scoring system to precisely record the time and duration of somatic signs and compare results among independent scorers to confirm that our criteria to identify the relevant behaviors (*i.e.* grooming, scratching, genital licking, shaking, twitching or jumping) was consistent. While we did observe an effect of nicotine precipitation on somatic signs compared to acute saline injection (non-precipitating), under our baseline conditions nicotine-exposed animals did not exhibit a statistically significant change in the number or timing of somatic signs following precipitation of withdrawal compared with mecamylamine-injected saline-exposed animals. Based on available literature, we are puzzled by the lack of an increase in somatic signs in chronic nicotine-exposed mice following mecamylamine-induced withdrawal. Our experimental paradigm is clearly sensitive to detect such changes, given that chemogenetic activation of the Amigo1 neurons was sufficient to elicit a significant decrease in somatic signs. It’s possible that specific experimental parameters unique to each laboratory may produce unique influences on the outcome of each study type. Variables such as animal strain and husbandry practices, prior handling by the behavioral technician, pre-exposure to the behavioral environment, room illumination conditions and prior acclimatization to i.p. injections may influence the overall anxiety/stress level of the mice. These may also translate to carry-through effects on the behavior outcomes, such as open arm times in the affective tests or outward expression of physical discomfort in somatic evaluations. On the other hand, we would like to highlight that we omitted digging behaviors (by not providing any substrate in the arena) as this behavior may overlap with the affective phenotype, given the established association of marble-burying with anxiogenesis during nicotine withdrawal (Chellian et al., 2021; Zhao-Shea et al., 2015). Indeed, whether many behaviors often quantified as somatic signs are indicators of physical distress or are instead additional manifestations of negative affect sharing the same neurobiological mechanisms remains elusive. Taken together, our results indicate that affective and somatic signs are separable components of the nicotine withdrawal phenotype that are dissociable at the behavioral level in both male and female mice. Of note, we also found that hM4Di-mediated inhibition of Amigo1 IPN neurons in saline-exposed mice elicited a small but distinct effect in males and females in one subtype of somatic signs (Supplementary Fig. 13D). Future studies are warranted to reveal the extent to which the IPN could contribute to some of the sex differences observed in somatic signs (Chellian et al., 2021).

By separately manipulating Amigo1 and Epyc GABAergic populations within the IPN, we have demonstrated that somatic and affective signs of withdrawal are separable phenomena at the behavioral level. Specifically, chemogenetic stimulation of Amigo1-IPN neuron activity reduced the number and time of somatic signs in nicotine-naïve mice, an effect that is opposite to the nicotine withdrawal phenotype. Stimulation of Amigo1-IPN neurons also increased locomotor activity but did not alter the pattern of exploration of the arena (Supplementary Fig. 9). Manipulation of Amigo1-IPN thus does not appear to mimic the overall withdrawal phenotype, although we cannot rule out that changes to locomotion may partially underlie observation of somatic signs, which are typically exhibited when animals are stationary. Moreover, we did observe some scattered local uptake of AAV just outside the IPN in Amigo1-cre animals, such that we cannot rule out scattered off-target activity as a caveat to some of our behavior assays. However, we consider it more likely that Amigo1-IPN neurons lie at the intersection of two related but different circuits underlying the physical and affective components of withdrawal, which may integrate different relevant afferent information (*e.g.* from the medial habenula, medial raphe nucleus or putative dopaminergic inputs) (DeGroot et al., 2020; Lima et al., 2017; Quina et al., 2017). Other manipulations of IPN neuron activity, such as optogenetic stimulation of GAD2+ neurons, differentially impact these two behavioral domains by increasing somatic signs while not eliciting any changes in anxiety-like behavior (Zhao-Shea et al., 2013). However, a similar manipulation has been shown to reduce anxiety-like behavior in animals undergoing spontaneous withdrawal (Klenowski et al., 2022). In addition to differences associated with different models of nicotine exposure (constant subcutaneous delivery or intermittent oral consumption) and withdrawal (precipitated or spontaneous), our findings reveal the need for subpopulation-specific studies of the mechanisms underlying nicotine withdrawal, and underscore the value of the Amigo1-cre line as a tool to probe those distinct phenotypes. Moreover, while our study relied on the elevated plus maze model as a proxy for anxiety with high construct validity, future studies may benefit from a broader interpretation of the anxiety phenotype using large batteries of orthogonal behavior tests to parse the contributions of Amigo1 (or Epyc) neurons to separate components of anxiety-like behavior. Similarly, the presence of an overlapping locomotor phenotype involving these populations, which manifests in both changes to maze exploration and apparent arousal, is a key consideration in any interpretation of behavioral outcomes in mice receiving experimental manipulation of activity in the IPN.

We cannot rule out that Epyc neurons may play a role in anxiogenesis under different conditions not revealed through our experimental paradigm; however their contribution appears from our data to be less robust or more selective than the large effects observed with manipulation of Amigo1 neuron activity. Moreover, Epyc stimulation appeared to impact locomotor activity to a larger extent than it did in Amigo1 neurons (Supplementary Figs. 9–10). This finding suggests that, insomuch that there is probable overlap between the influence of IPN neuronal activity on locomotor and withdrawal behaviors, these two subpopulations may contribute differently to each of them. The main source of glutamatergic and cholinergic innervation to both of these GABAergic subpopulations in the IPN is the medial habenula (Ables et al., 2017; Frahm et al., 2015; Ren et al., 2011) and the IPN is reciprocally connected to brain structures associated with anxiety such as the laterodorsal tegmentum and the serotonergic Raphe nuclei (Lima et al., 2017; Quina et al., 2017). The ventral tegmental area could be another key input to the IPN, but this projection remains controversial (DeGroot et al., 2020; Quina et al., 2017). Our findings therefore indicate that to further elucidate how IPN drives the withdrawal phenotype, it will be necessary to account for both its internal and external connectivity. Future studies of how nicotine exposure drives plasticity in the different IPN synapses will further differentiate these phenotypes. In this sense, an important future objective is to identify and characterize the different circuitry engaged up and downstream the Amigo1 subpopulation of IPN neurons, both in the context of understanding and treating nicotine withdrawal and in leveraging the contribution of this circuit to general anxiety phenotypes.

## Supplementary Material

1

## Data Availability

Data will be made available on request.

## Appendix A. Supplementary data

Supplementary data to this article can be found online at https://doi.org/10.1016/j.nbd.2025.107240.
