# Supplementary material for: Chemogenetic control of GABAergic neurons within the interpeduncular nucleus reveals dissociable behavioral components of the nicotine withdrawal phenotype
Source: Neurobiol Dis. Author manuscript; Available in PMC 2026 Jul 9. (PMC13348565; doi:10.1016/j.nbd.2025.107240)
Supplement: 1 [file NIHMS2188790-supplement-1.pdf]

Supplementary Materials
(Supplementary Figures 1-16 and Tables 1-6)

Chemogenetic control of GABAergic neurons within the interpeduncular nucleus reveals dissociable behavioral components of the nicotine withdrawal phenotype

Running title: Activity of interpeduncular neurons regulates nicotine withdrawal behaviors

Anabel M. M. Miguelez Fernández (PhD), Shana Netherton (MSc), Seshadri B. Niladhuri (MD), Alexander C. Brown (BSc), Patricia Rivera (BSc), Kuei Y. Tseng (MD, PhD) and Christian J. Peters (PhD)*

Department of Anatomy and Cell Biology, University of Illinois Chicago – College of Medicine, Chicago, Illinois, 60612, USA

Suppl_Figure_1_IPN paper
Miguelez Fernández et al. 2025

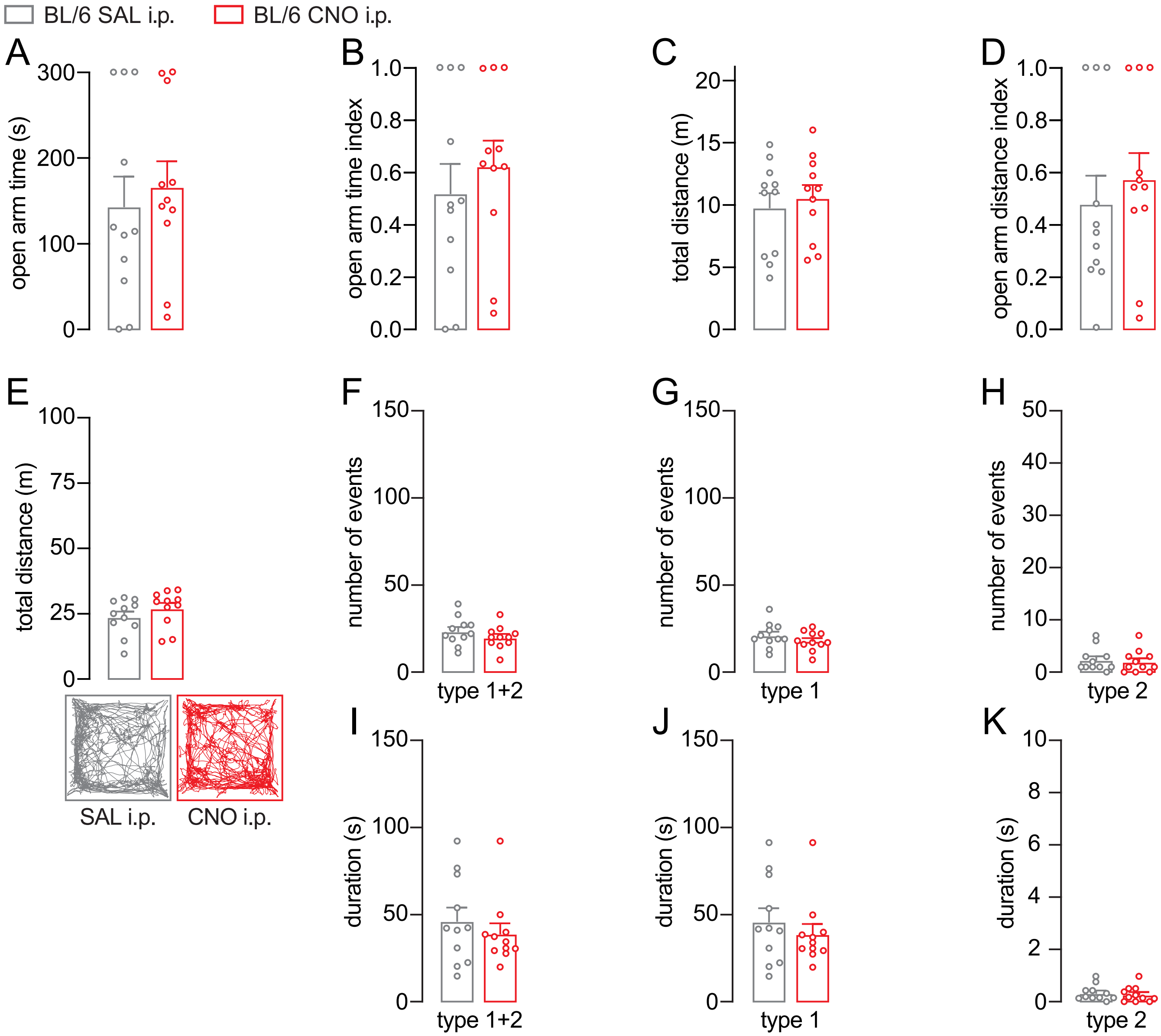

Supplementary Figure 1. Acute CNO injection does not alter anxiety-like or somatic behaviors. Mice of the background strain BL/6 received an i.p. injection of saline (SAL) or 1 mg/kg Clozapine-N-Oxide (CNO) 30 min before the start of testing. Experimental groups are as follows: BL/6 SAL i.p. (N=11), BL/6 CNO i.p. (N=11). **A-D)** Elevated plus maze. All mice presented a similar pattern of exploration of the maze as reflected by open arm time (**A:** p=0.62), open arm time index (**B:** p=0.49) and open arm distance index (**D:** p=0.53) (full details in *Methods*). No differences were observed in the total distance travelled in the maze (**C:** p=0.62). **E-K)** Immediately after the EPM test, mice were placed in an open arena and their behavior was evaluated for 15 minutes. **E)** *Top*: The total distance travelled in the arena was included as a measure of locomotor activity (p=0.27). *Bottom*: Example track plot for each group. **F-K)** Somatic signs. Behaviors were grouped in two types: 1) grooming-related (**G, J**) and 2) shakes & twitches (**H, K**) (full description in *Methods*). No changes were observed between groups regarding the total number (**F:** p=0.27) and duration (**I:** p=0.46) of somatic behaviors. Similarly, no changes were observed in number or duration of type 1 (**G:** p=0.25; **J:** p=0.46) and type 2 events (**H:** p=0.70; **K:** p=0.64). Two-tailed, unpaired t-test.

Suppl_Figure 2_IPN paper
Miguelez Fernández et al. 2025

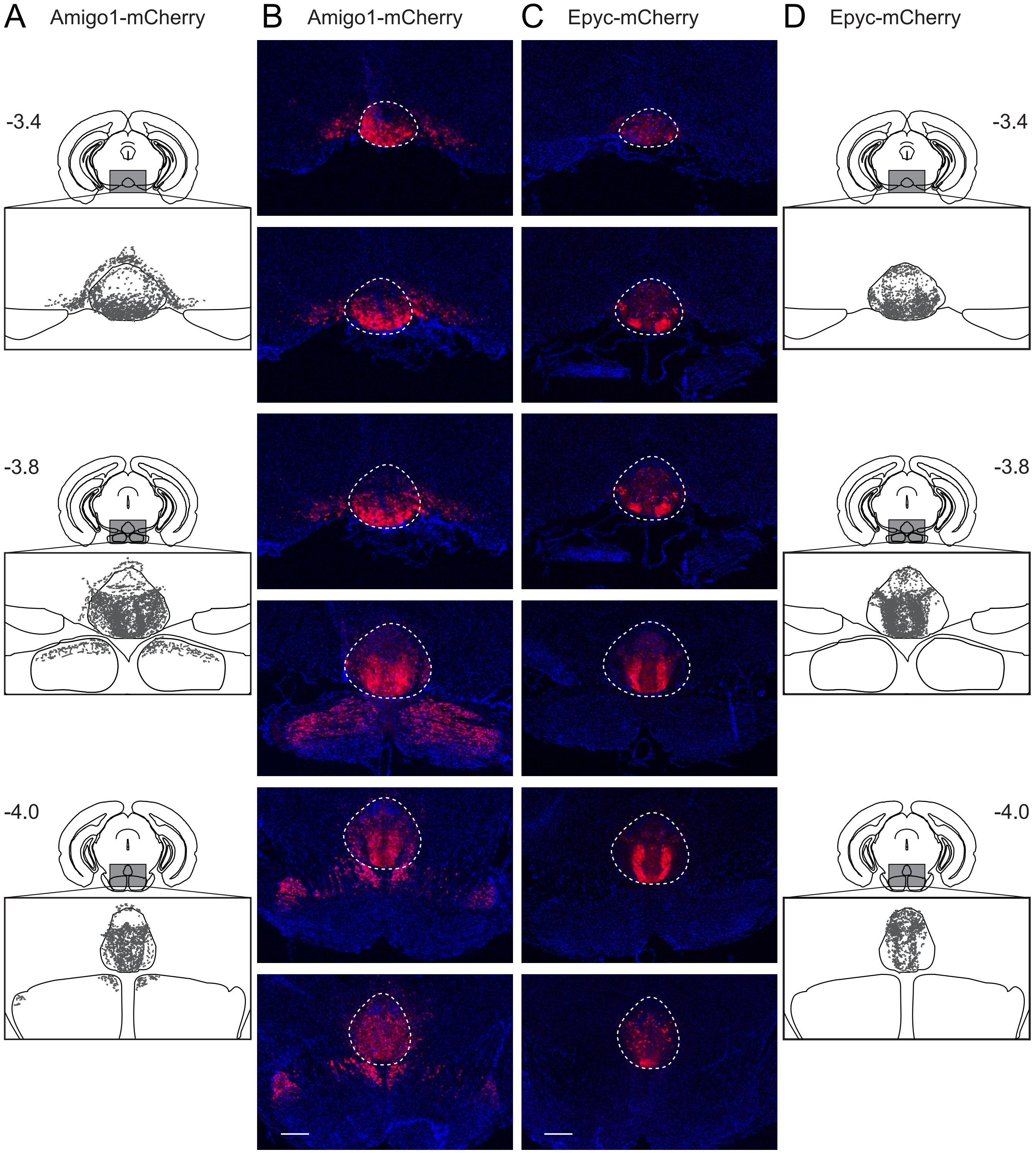

Supplementary Figure 2. Pattern of virus expression in Amigo1-cre and Epyc-cre mice. Schematic representation summarizing the extent of virus expression in Amigo1-cre **(A)** and Epyc-cre **(D)** mice injected with AAV9-hSyn-DIO-mCherry. Numbers indicate mm relative to bregma. **B-C)** Representative images (~120µm apart) of the virus expression pattern in the IPN (outlined) of Amigo1-cre and Epyc-cre are shown from anterior (top) to posterior (bottom). Scale bars are 250 µm and apply to all images.

Suppl_Figure_3_IPN paper
Miguelez Fernández et al. 2025

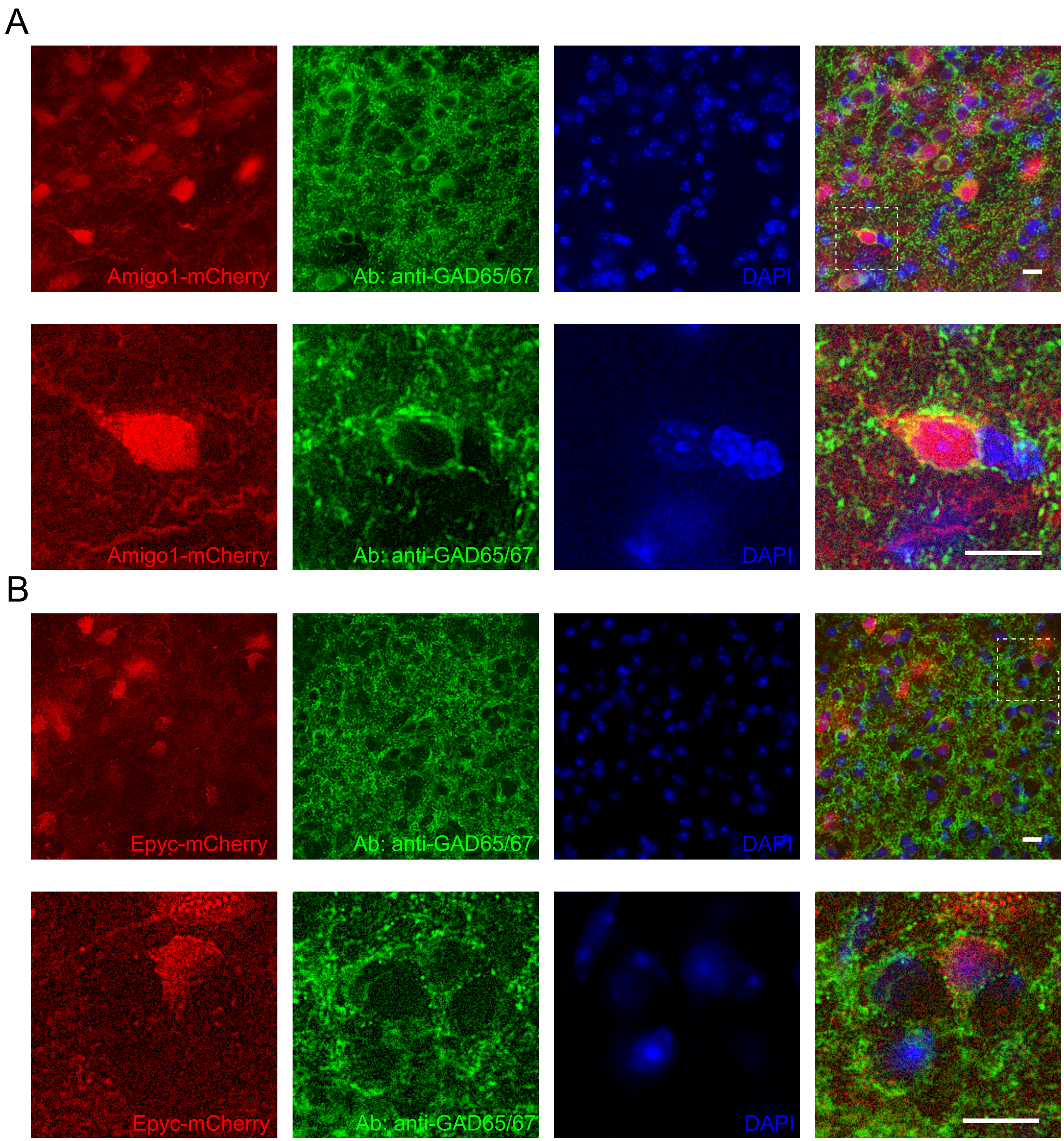

Supplementary Figure 3. Amigo1 and Epyc neurons are labeled by an antibody against GABAergic marker GAD65/67. A) Epifluorescence images of cryosections from Amigo1-cre mice expressing AAV-DIO-mCherry (red) in IPN neurons, stained with anti-GAD65/67 (green) and DAPI (blue). The bottom row shows an inset view from the dashed box in the merged image. Scale bars represent 10 μm and apply to the entire row. **B)** Epifluorescence images of cryosections from Epyc-cre mice expressing AAV-DIO-mCherry (red) in IPN neurons, stained with anti-GAD65/67 (green) and DAPI (blue). The bottom row shows an inset view from the dashed box in the merged image. Scale bars represent 10 μm and apply to the entire row. Images from each genotype are representative of at least 3 separate staining experiments.

Suppl_Figure_4_IPN paper
Miguelez Fernández et al. 2025

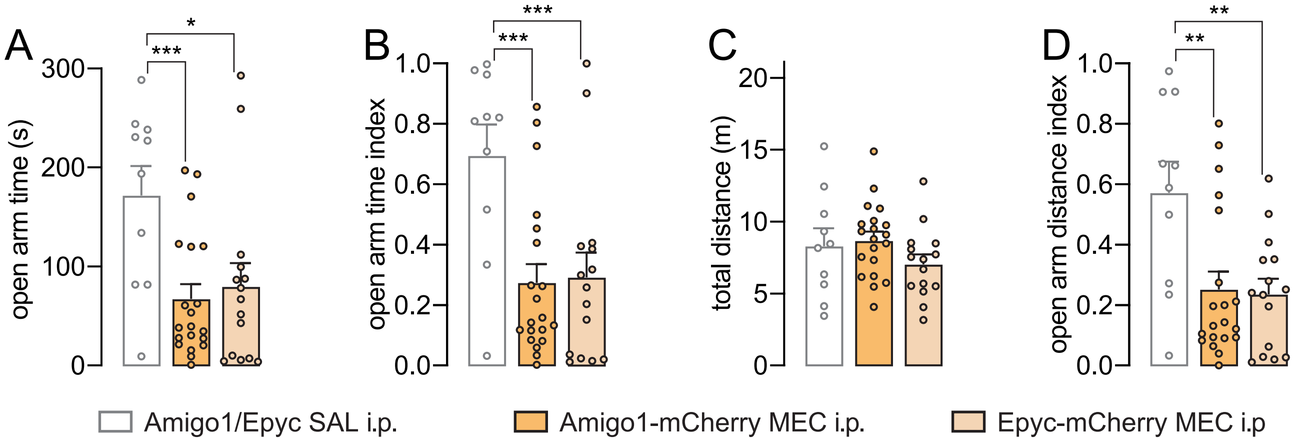

Supplementary Figure 4. Chronic exposure to nicotine increases anxiety-like behavior in mice following mecamylamine injection. All mice were chronically exposed to nicotine for 14 days via s.c. implantation of osmotic pumps. On the day of the test, mice received an i.p. injection of saline (SAL) or 3 mg/kg of mecamylamine (MEC) 5 min before the start of testing. Experimental groups are as follows: Amigo1/Epyc SAL i.p. (N=10), Amigo1-mCherry MEC i.p. (N=20), Epyc-mCherry MEC i.p. (N=15). **A-D)** Elevated plus maze. Animals that received mecamylamine i.p. presented reduced open arm time (**A:** $F_{(2,42)}$=6.37, p=0.004) with respect to their saline injected counterparts. This anxiety-like behavior was also reflected by a reduction in their open arm time (**B:** $F_{(2,42)}$=8.00, p=0.001) and distance (**D:** $F_{(2,42)}$=6.79, p=0.003) indices (full details in *Methods*). No changes were observed in the total distance travelled in the maze (**C:** $F_{(2,42)}$=1.51, p=0.23). One-way ANOVA, *p<0.05, **p<0.01, ***p<0.005.

Suppl_Figure_5_IPN paper
Miguelez Fernández et al. 2025

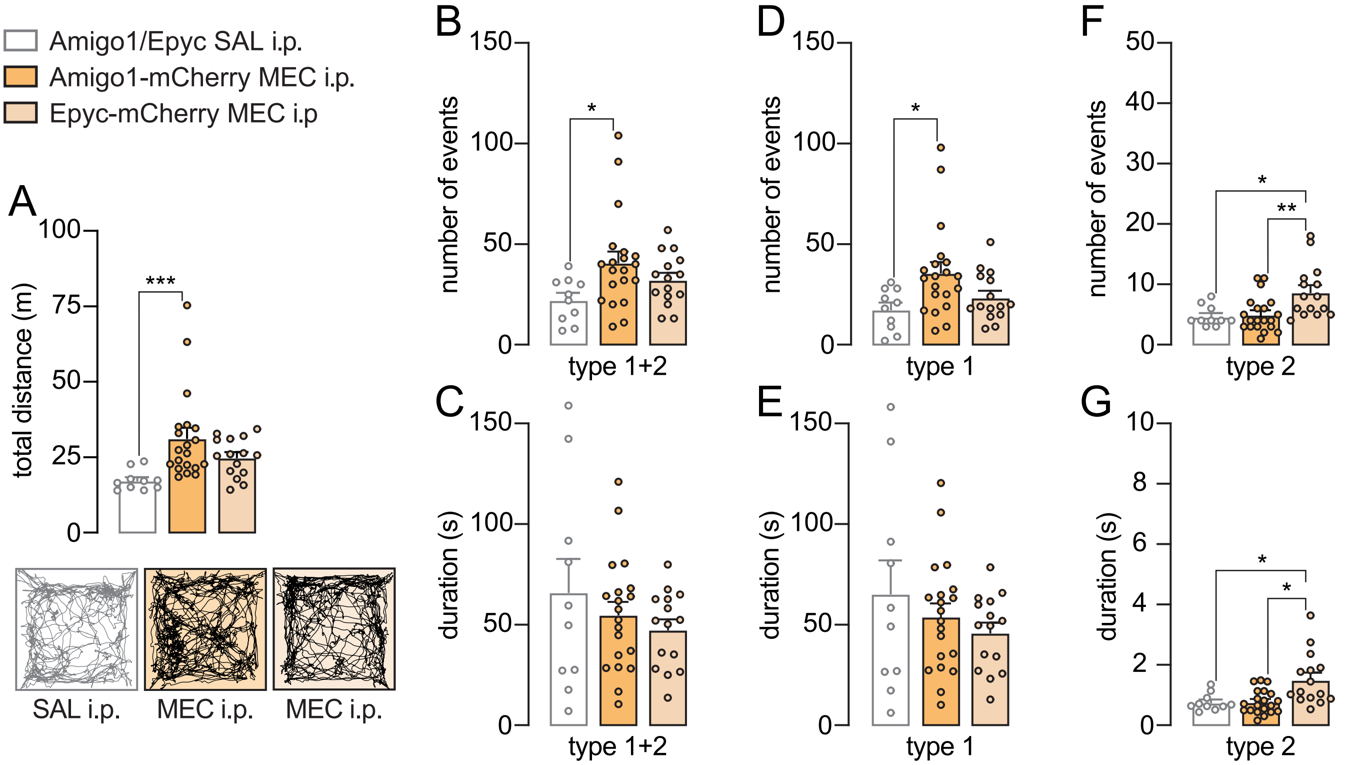

Supplementary Figure 5. Effect of chronic exposure to nicotine on somatic behaviors following mecamylamine injection. A) *Top*: The total distance travelled in the arena was included as a measure of locomotor activity ($F_{(2,42)}$=5.81, p=0.006). *Bottom*: Example track plot for each group. **B-G)** Somatic signs. Behaviors were grouped in two types: 1) grooming-related (**D, E**) and 2) shakes & twitches (**F, G**) (full description in *Methods*). No changes were observed between groups regarding the total number (**B:** $F_{(2,42)}$=3.49, p=0.04) and duration (**C:** $F_{(2, 15.39)}$=0.74, p=0.49) of somatic behaviors. In contrast to Epyc-mCherry animals, the number of type 1 events was significantly larger for Amigo1-mCherry mice that received mecamylamine i.p. compared with saline injected controls (**D:** $F_{(2, 42)}$=4.19, p=0.002). No changes were observed in the duration of type 1 behaviors (**E:** $F_{(2, 15.32)}$=0.81, p=0.46). Notably, Epyc-mCherry animals that received mecamylamine injection displayed a higher number (**F:** $F_{(2, 42)}$=6.81, p=0.003) and duration (**G:** $F_{(2, 22.58)}$=8.60, p=0.002) of type 2 behaviors with respect to their Amigo and saline injected counterparts. One-way ANOVA, *p<0.05, **p<0.01, ***p<0.005.

Suppl_Figure_6_IPN paper
Miguelez Fernández et al. 2025

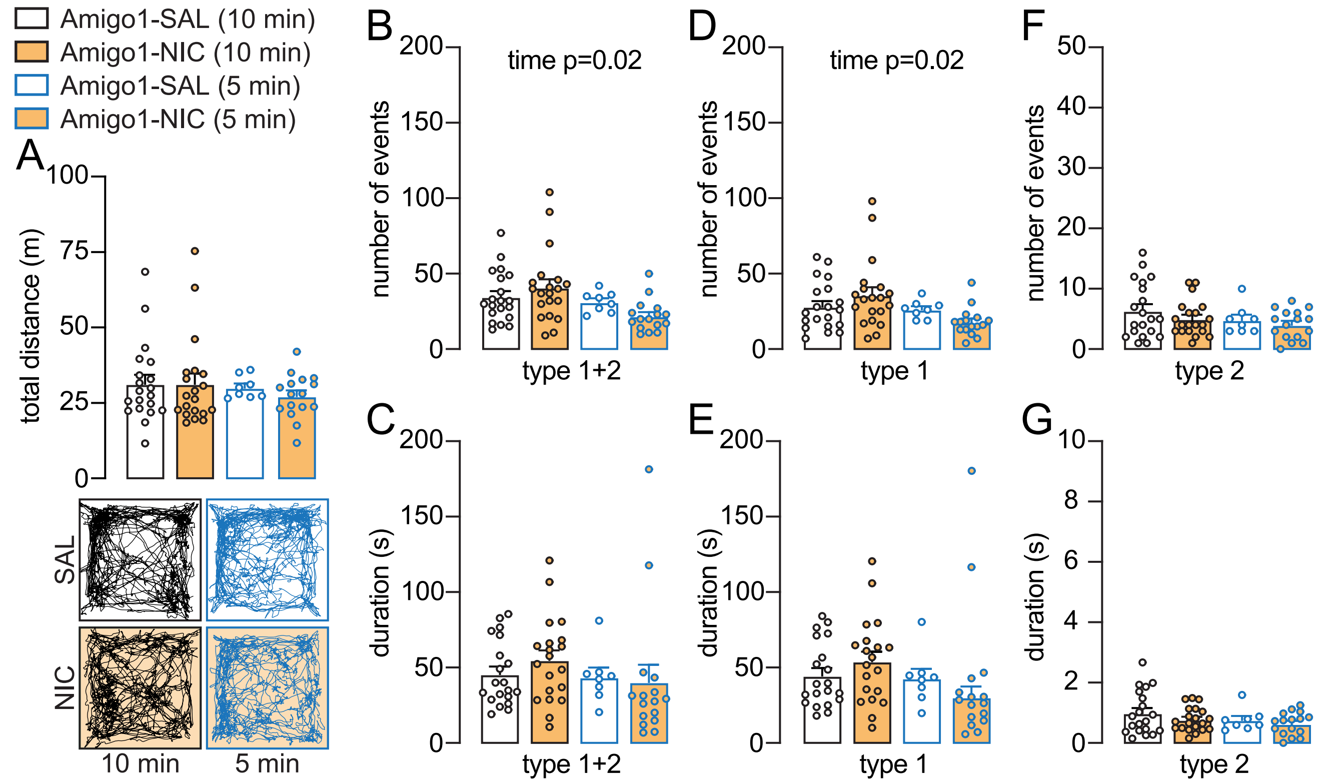

Supplementary Figure 6. Somatic behaviors at 5 or 10 minutes after mecamylamine injection in mice chronically exposed to nicotine. A separate cohort of Amigo1-mCherry animals was generated to test whether any changes in the somatic signs of nicotine exposed animals were observed at a shorter time after mecamylamine injection (i.e. at 5 minutes post injection, when effects on anxiety-like behavior were present as shown in Figure 1). Comparisons are made with Amigo1-mCherry NIC animals tested after completing the EPM (as shown in Figure 3). **A)** *Top*: Total distance travelled in the arena was similar for all experimental groups (treatment x time $F_{(1,60)}$=0.20, p=0.65; treatment $F_{(1,60)}$=0.19, p=0.66; time $F_{(1,60)}$=0.71, p=0.40). *Bottom*: Example track plot for each group. **B-G)** Somatic signs. Behaviors were grouped in two types: 1) grooming-related (**D-E**) and 2) shakes & twitches (**F-G**) (full description in *Methods*). No effect of treatment was evidenced for the total number (**B:** treatment x time $F_{(1,60)}$=2.76, p=0.10; treatment $F_{(1,60)}$=0.11, p=0.74; time $F_{(1,60)}$=5.75) or duration of these somatic behaviors (**C:** treatment x time F($_{1,60)}$=0.57, p=0.46; treatment $F_{(1,60)}$=0.13, p=0.72; time $F_{(1,60)}$= 1.00, p=0.32). Similarly, nicotine treatment did not change the number (**D:** treatment x time $F_{(1,60)}$=3.28, p=0.11; treatment $F_{(1,60)}$=0.01, p=0.92; time $F_{(1,60)}$=5.03) or duration (**E:** treatment x time $F_{(1,60)}$=0.58, p=0.45; treatment $F_{(1,60)}$=0.15, p=0.70; time $F_{(1,60)}$=0.96, p=0.33) of type 1 or type 2 events (**F:** treatment x time $F_{(1,60)}$=0.09, p=0.77; treatment $F_{(1,60)}$=1.41, p=0.21; time $F_{(1,60)}$=1.90, p=0.17; **G:** treatment x time $F_{(1,60)}$=0.12, p=0.73; treatment $F_{(1,60)}$=1.59, p=0.21; time $F_{(1,60)}$=1.82, p=0.18). Two-way ANOVA.

Suppl_Figure_7_IPN paper
Miguelez Fernández et al. 2025

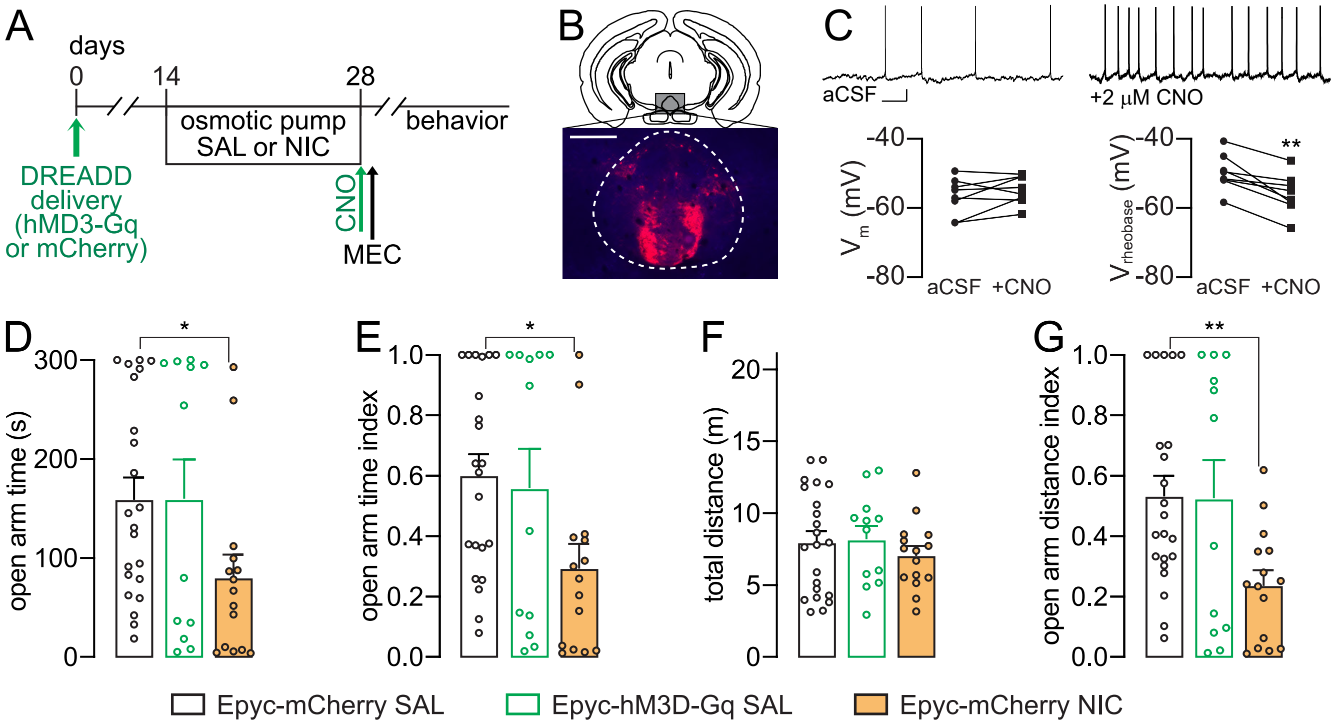

Supplementary Figure 7. Stimulation of Epyc-IPN neurons does not alter anxiety-like behaviors in nicotine-naïve mice. A) Timeline of the experimental design. Experimental groups are as follows: Epyc-mCherry SAL (N=22), Epyc-hM3D-Gq SAL (N=12) and Epyc-mCherry NIC (N=15). **B)** Schematic diagram of virus injection target and representative image of virus expression in the IPN (outlined) of Epyc-cre male and female adult mice. Scale bar: 250µm **C)** *Ex-vivo* electrophysiology. *Top*: Sample traces of action potentials from Epyc-cre cells expressing hM3D-Gq in response to aCSF alone or supplemented with 2µM CNO. Scale bar indicates 10mV and 500ms and applies to all traces. *Bottom*: A negative shift in the rheobase for action potential voltage (f<0.1 Hz) from -49.9 ± 1.8 mV to -56.0 ± 2.0 mV was observed following CNO application (*right*) (n=8, p=0.002, t-test) but no change in resting membrane potential was seen which was -56.7 ± 1.9 mV before and -54.8 ± 1.4 mV after CNO application (*left*) (n=8, p=0.21, t-test). **D-G)** Elevated plus maze. Maze exploration was assessed by evaluating time spent in the open arms (**D:** $F_{(2,24.87)}$=3.51, p=0.05) and the total distance travelled in the maze (**F:** $F_{(2,46)}$=0.48, p=0.62) as a measure of locomotor activity. In addition, an open arm index was calculated for time spent (**E:** $F_{(2,24.63)}$=4.40, p=0.02) and distance travelled (**G:** $F_{(2,23.61)}$=4.06, p=0.03) (full details in *Methods*). One-way ANOVA. *p<0.05, **p<0.01, ***p<0.005.

Suppl_Figure_8_IPN paper
Miguelez Fernández et al. 2025

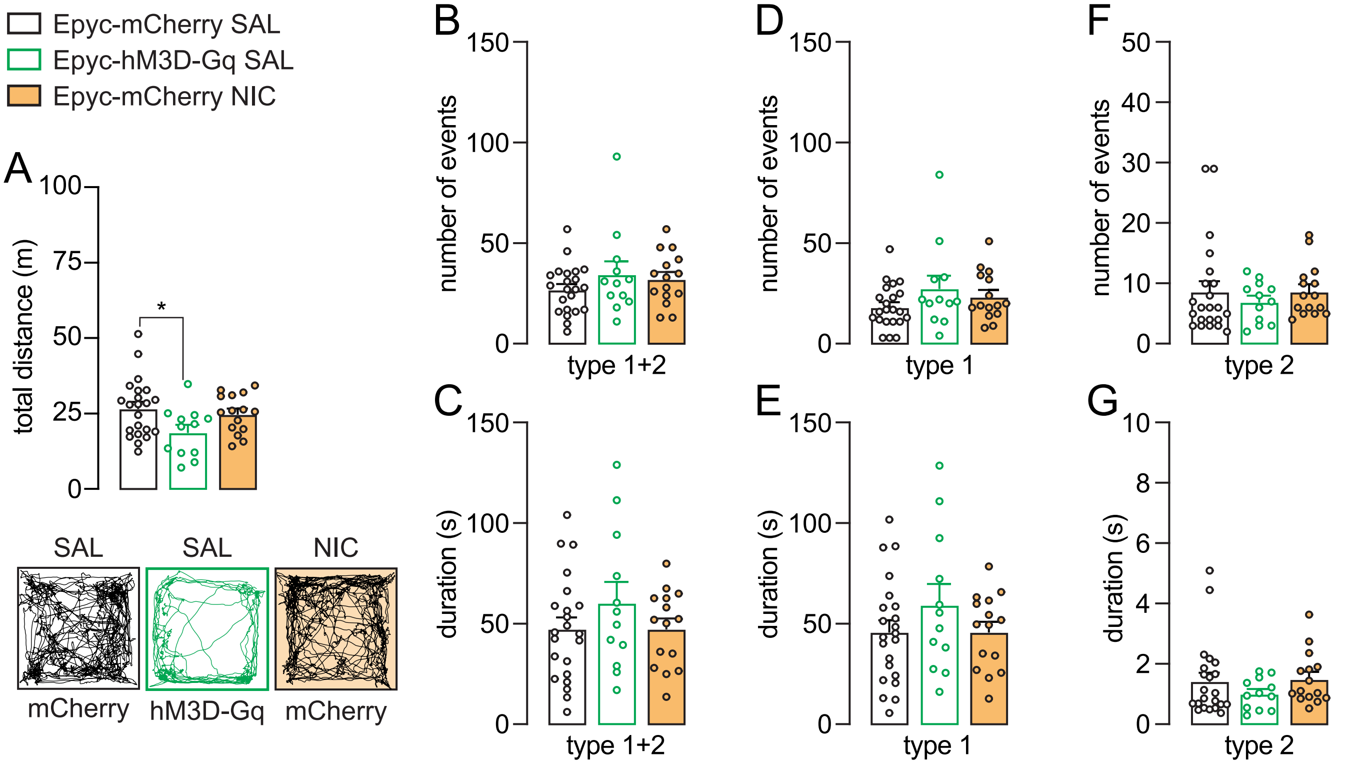

Supplementary Figure 8. Stimulation of Epyc-IPN neurons does not alter somatic behaviors in nicotine-naïve mice but reduces overall locomotor activity. A) *Top*: Total distance travelled in the arena was significantly smaller for the Epyc-hM3D-Gq group ($F_{(2,46)}$=3.53, p=0.04) *Bottom*: Example track plot for each group. **B-G)** Somatic signs. Behaviors were grouped in two types: 1) grooming-related (**D, E**) and 2) shakes & twitches (**F, G**) (full description in *Methods*). No changes were observed in the total number of somatic behaviors (**B:** $F_{(2,46)}$=1.12, p=0.34) or their duration (**C:** $F_{(2,46)}$=1.06, p=0.36) following stimulation of Epyc-IPN neurons. Similarly, the number (**D:** $F_{(2, 46)}$=1.70, p=0.19) and duration (**E:** $F_{(2,46)}$=1.14, p=0.33) of type 1 and type 2 events (**F:** $F_{(2,46)}$=0.38, p=0.69; **G:** $F_{(2,46)}$=0.98, p=0.38) remained unaltered. One-way ANOVA. *p<0.05, **p<0.01, ***p<0.005.

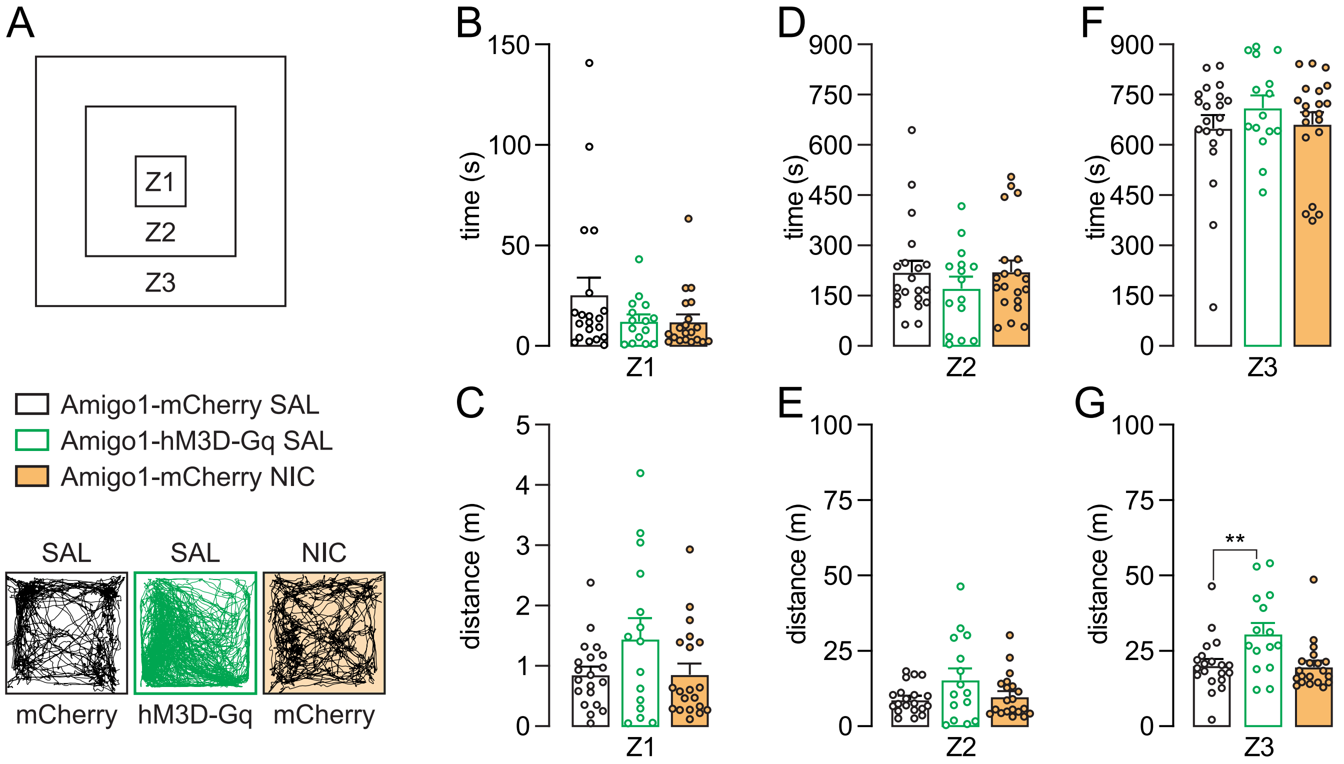

Supplementary Figure 9. Stimulation of Amigo1-IPN neurons does not significantly change the exploration pattern of the arena during somatic behavior assessment. A) *Top:* Schematic diagram of three zones (Z1, Z2, Z3) in the arena. *Bottom:* Example track plot of the exploration pattern for each group (as presented in main Figure 6A). **B-G)** Exploration of the field was similar for all groups as revealed by the occupancy time and distance travelled in Z1 (**B**: $F_{(2,52)}$=1.87, p=0.16; **C**: $F_{(2,27.25)}$=2.16, p=0.13), Z2 (**D**: $F_{(2,52)}$=0.69, p=0.50; **E**: $F_{(2,24.09)}$=2.22, p=0.13) as well as the time spent in Z3 (**F**: $F_{(2,52)}$=0.73, p=0.49). The distance travelled in Z3 of the arena was significantly higher for the Amigo1-hM3D-Gq group (**G**: $F_{(2,52)}$=6.29, p=0.004) One-way ANOVA.*p<0.05, **p<0.01, ***p<0.005.

Suppl_Figure 10_IPN paper
Miguelez Fernández et al. 2025

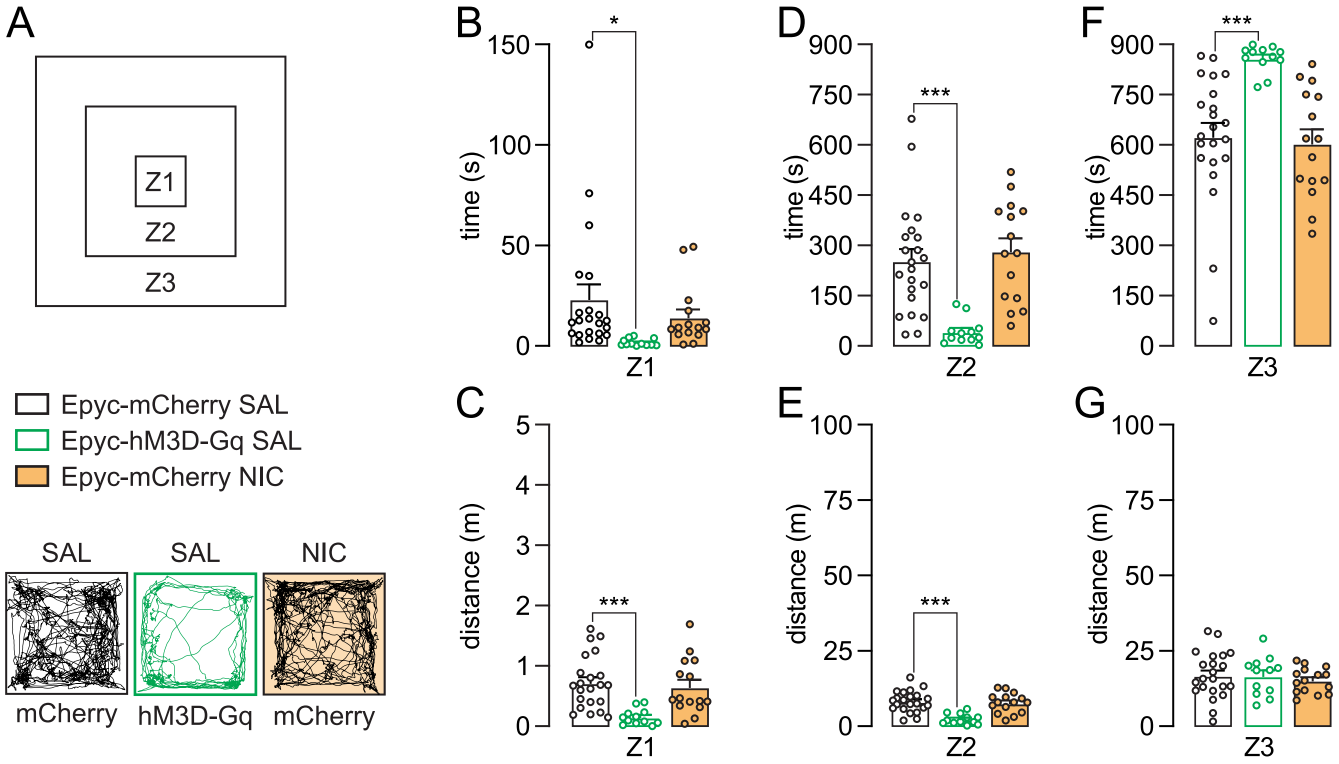

Supplementary Figure 10. Stimulation of Epyc-IPN neurons changes the exploration pattern of the arena during somatic behavior assessment. A) *Top:* Schematic diagram of three zones (Z1, Z2, Z3) in the arena. *Bottom:* Example track plot of the exploration pattern for each group (as presented in Suppl. Figure 8a). **B-G)** Exploration of the field was altered following stimulation of Epyc-IPN neurons such that the occupancy time of Z1
(**B**: $F_{(2,30.7)}$=4.63, p=0.02) and Z2 (**D**: $F_{(2,35.6)}$=14.65, p<0.0001) was significtly reduced, while the time spent in Z3 (**F**: $F_{(2,36.42)}$=13.50, p<0.0001) was increased. Changes in the total distance (shown in Suppl. Figure 8a.) are due to reduced distance travelled in Z1 (**C**: $F_{(2,33.74)}$=10.00, p=0.0004) and Z2 (**E**: $F_{(2,46)}$=15.24, p<0.0001) since distance travelled in Z3 of the arena was similar for all groups (**G**: $F_{(2,46)}$=0.32, p=0.7266). One-way ANOVA.*p<0.05, **p<0.01, ***p<0.005.

Suppl_Figure 11_IPN paper
Miguelez Fernández et al. 2025

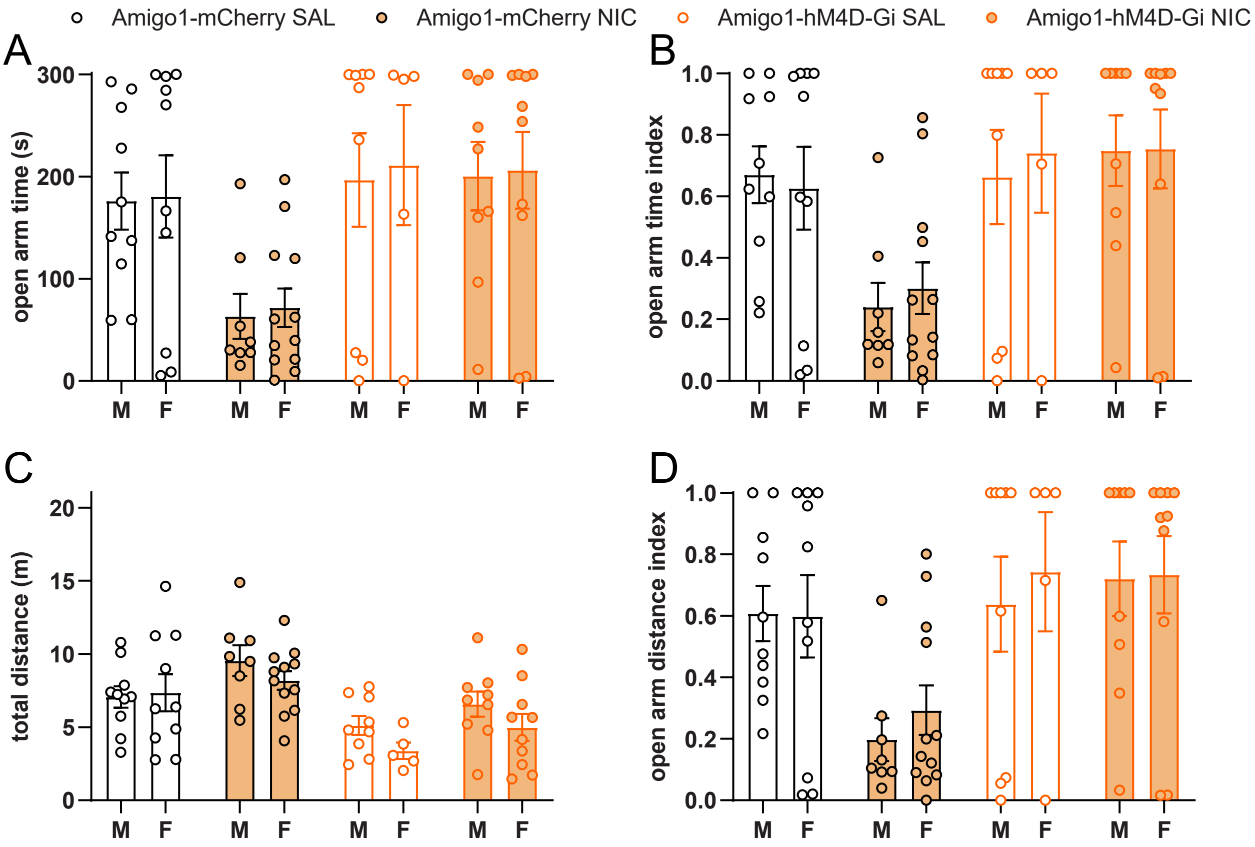

Supplementary Table 1: Three-way ANOVA analysis of main Figure 1 data including sex as a factor

	**A** (re:Figure 1D)		**B** (re:Figure 1E)		**C** (re:Figure 1F)		**D** (re:Figure 1G)	
Factor	F (DFn, DFd)	p value	F (DFn, DFd)	p value	F (DFn, DFd)	p value	F (DFn, DFd)	p value
sex	F (1, 65) = 0.11	0.75	F (1, 65) = 0.08	0.77	F (1, 65) = 2.83	0.10	F (1, 65) = 0.34	0.56
sex x virus	F (1, 65) = 0.006	0.94	F (1, 65) = 0.04	0.85	F (1, 65) = 0.75	0.39	F (1, 65) = 0.008	0.93
sex x treatment	F (1, 65) = 0.002	0.96	F (1, 65) = 0.009	0.92	F (1, 65) = 0.33	0.57	F (1, 65) = 0.001	0.97
sex x virus x treatment	F (1, 65) = 0.02	0.90	F (1, 65) = 0.25	0.62	F (1, 65) = 0.47	0.50	F (1, 65) = 0.32	0.57

Suppl_Figure 12_IPN paper
Miguelez Fernández et al. 2025

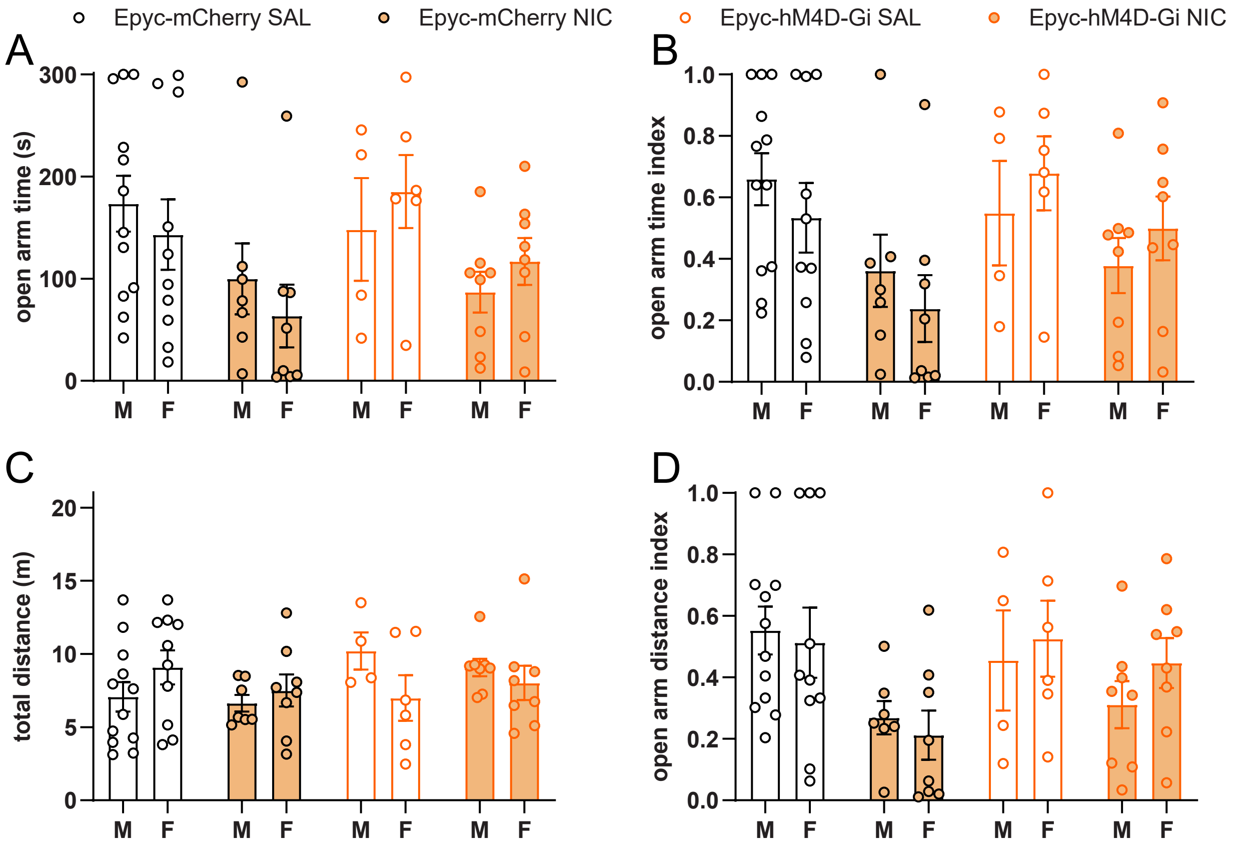

Supplementary Table 2: Three-way ANOVA analysis of main Figure 2 data including sex as a factor

	**A** (re:Figure 2D)		**B** (re:Figure 2E)		**C** (re:Figure 2F)		**D** (re:Figure 2G)	
Factor	F (DFn, DFd)	p value	F (DFn, DFd)	p value	F (DFn, DFd)	p value	F (DFn, DFd)	p value
sex	F (1, 55) = 0.00005	0.99	F (1, 55) =0.00007	0.99	F (1, 55) = 0.18	0.68	F (1, 55) = 0.15	0.70
sex x virus	F (1, 55) = 2.04	0.16	F (1, 55) = 2.37	0.13	F (1, 55) = 4.81	**0.03**	F (1, 55) = 1.14	0.29
sex x treatment	F (1, 55) = 0.02	0.89	F (1, 55) = 0.0004	0.99	F (1, 55) = 0.10	0.76	F (1, 55) = 0.03	0.87
sex x virus x treatment	F (1, 55) = 0.0001	0.99	F (1, 55) = 0.001	0.97	F (1, 55) = 1.02	0.32	F (1, 55) = 0.08	0.77
Bonferroni multiple comparisons test	N/A		N/A		p>0.05 for all comparisons		N/A	

Suppl_Figure 13_IPN paper
Miguelez Fernández et al. 2025

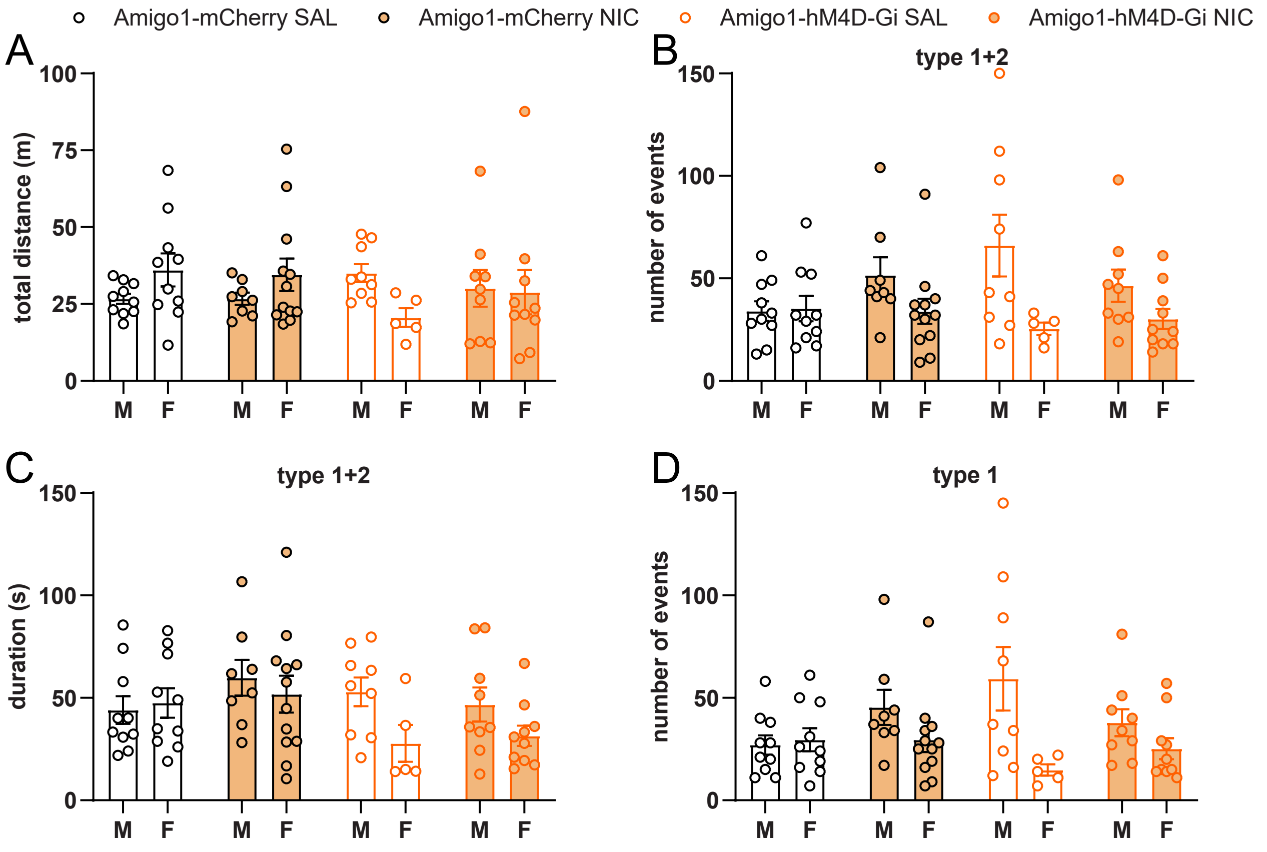

Supplementary Table 3A-D: Three-way ANOVA analysis of main Figure 3 data including sex as a factor

	**A** (re:Figure 3A)		**B** (re:Figure 3B)		**C** (re:Figure 3C)		**D** (re:Figure 3D)	
Factor	F (DFn, DFd)	p value	F (DFn, DFd)	p value	F (DFn, DFd)	p value	F (DFn, DFd)	p value
sex	F (1, 65) = 0.01	0.92	F (1, 65) = 9.84	**0.003**	F (1, 65) = 3.89	0.05	F (1, 65) = 9.47	**0.003**
sex x virus	F (1, 65) = 5.14	**0.03**	F (1, 65) = 3.01	0.09	F (1, 65) = 2.49	0.12	F (1, 65) = 3.70	0.06
sex x treatment	F (1, 65) = 0.64	0.43	F (1, 65) = 0.06	0.81	F (1, 65) = 0.005	0.95	F (1, 65) = 0.35	0.56
sex x virus x treatment	F (1, 65) = 1.04	0.31	F (1, 65) = 3.42	0.07	F (1, 65) = 0.88	0.35	F (1, 65) = 4.82	**0.03**
Bonferroni multiple comparisons test	p>0.05 for all comparisons		p>0.05 for all comparisons		N/A		Amigo1-Gi SAL M vs. Amigo1-Gi SAL F	**0.04**
							p>0.05 for all other comparisons	

Suppl_Figure 13_IPN paper
Miguelez Fernández et al. 2025

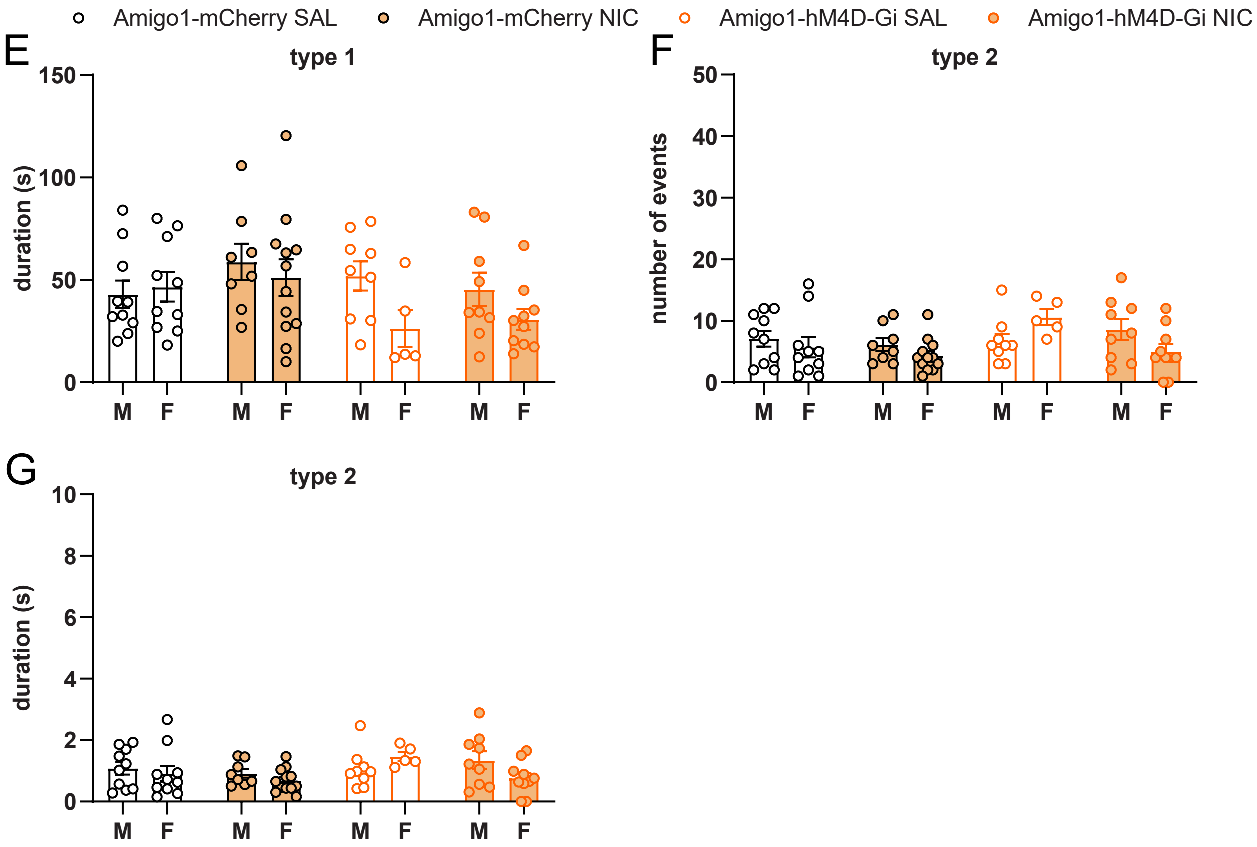

Supplementary Table 3E-G: Three-way ANOVA analysis of main Figure 3 data including sex as a factor

	**E** (re:Figure 3E)		**F** (re:Figure 3F)		**G** (re:Figure 3G)	
Factor	F (DFn, DFd)	p value	F (DFn, DFd)	p value	F (DFn, DFd)	p value
sex	F (1, 65) = 3.81	0.06	F (1, 65) = 0.54	0.46	F (1, 65) = 0.95	0.33
sex x virus	F (1, 65) = 2.54	0.12	F (1, 65) = 0.87	0.35	F (1, 65) = 0.18	0.67
sex x treatment	F (1, 65) = 0.0005	0.98	F (1, 65) = 4.26	**0.04**	F (1, 65) = 3.29	0.07
sex x virus x treatment	F (1, 65) = 0.96	0.33	F (1, 65) = 3.46	0.07	F (1, 65) = 2.54	0.12
Bonferroni multiple comparisons test	N/A		p>0.05 for all comparisons		N/A	

Suppl_Figure 14_IPN paper
Miguelez Fernández et al. 2025

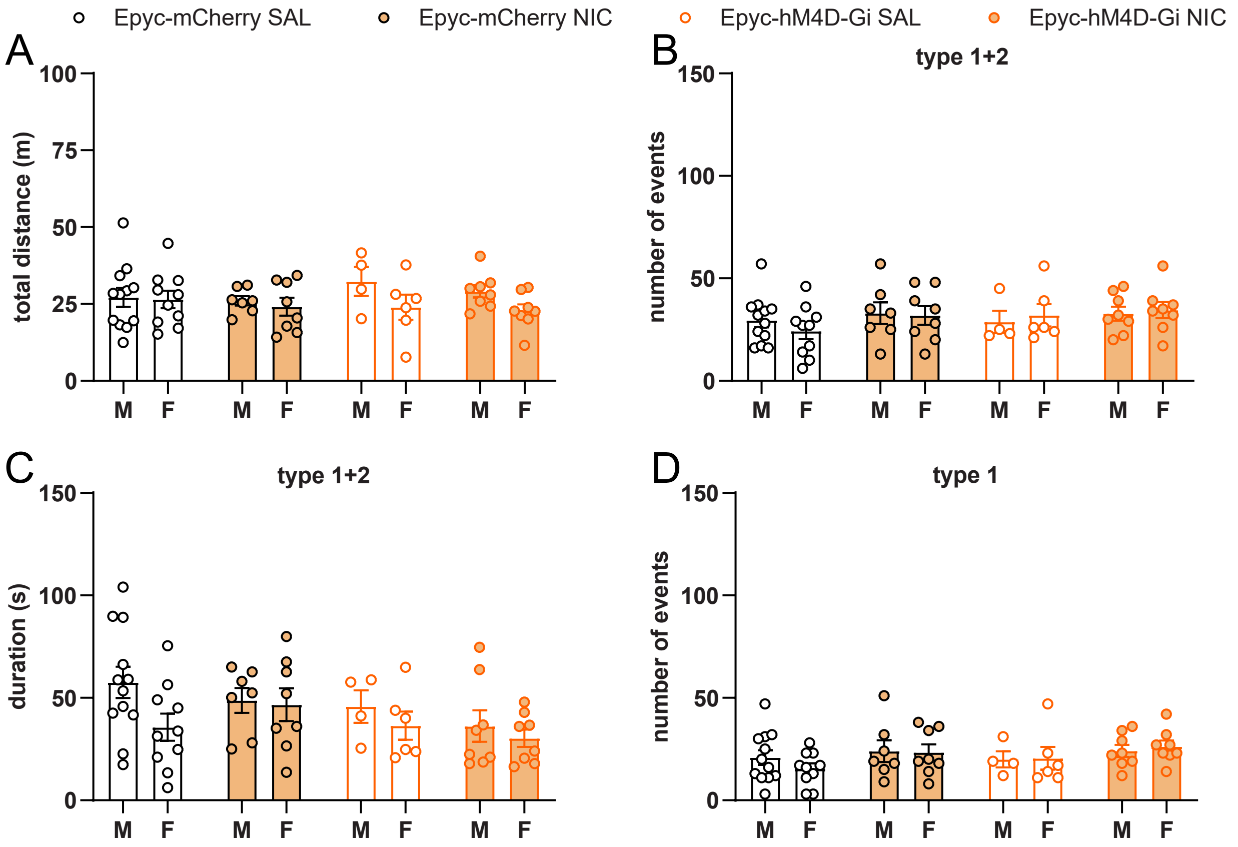

Supplementary Table 4A-D: Three-way ANOVA analysis of main Figure 4 data including sex as a factor

	**A** (re:Figure 4A)		**B** (re:Figure 4B)		**C** (re:Figure 4C)		**D** (re:Figure 4D)	
Factor	F (DFn, DFd)	p value	F (DFn, DFd)	p value	F (DFn, DFd)	p value	F (DFn, DFd)	p value
sex	F (1, 55) = 3.87	0.05	F (1, 55) = 0.01	0.92	F (1, 55) = 3.25	0.08	F (1, 55) = 0.09	0.76
sex x virus	F (1, 55) = 1.91	0.17	F (1, 55) = 0.79	0.38	F (1, 55) = 0.16	0.69	F (1, 55) = 0.59	0.45
sex x treatment	F (1, 55) = 0.006	0.94	F (1, 55) = 0.04	0.84	F (1, 55) = 1.15	0.29	F (1, 55) = 0.33	0.57
sex x virus x treatment	F (1, 55) = 0.14	0.71	F (1, 55) = 0.19	0.66	F (1, 55) = 0.56	0.46	F (1, 55) = 0.08	0.78

Suppl_Figure 14_IPN paper
Miguelez Fernández et al. 2025

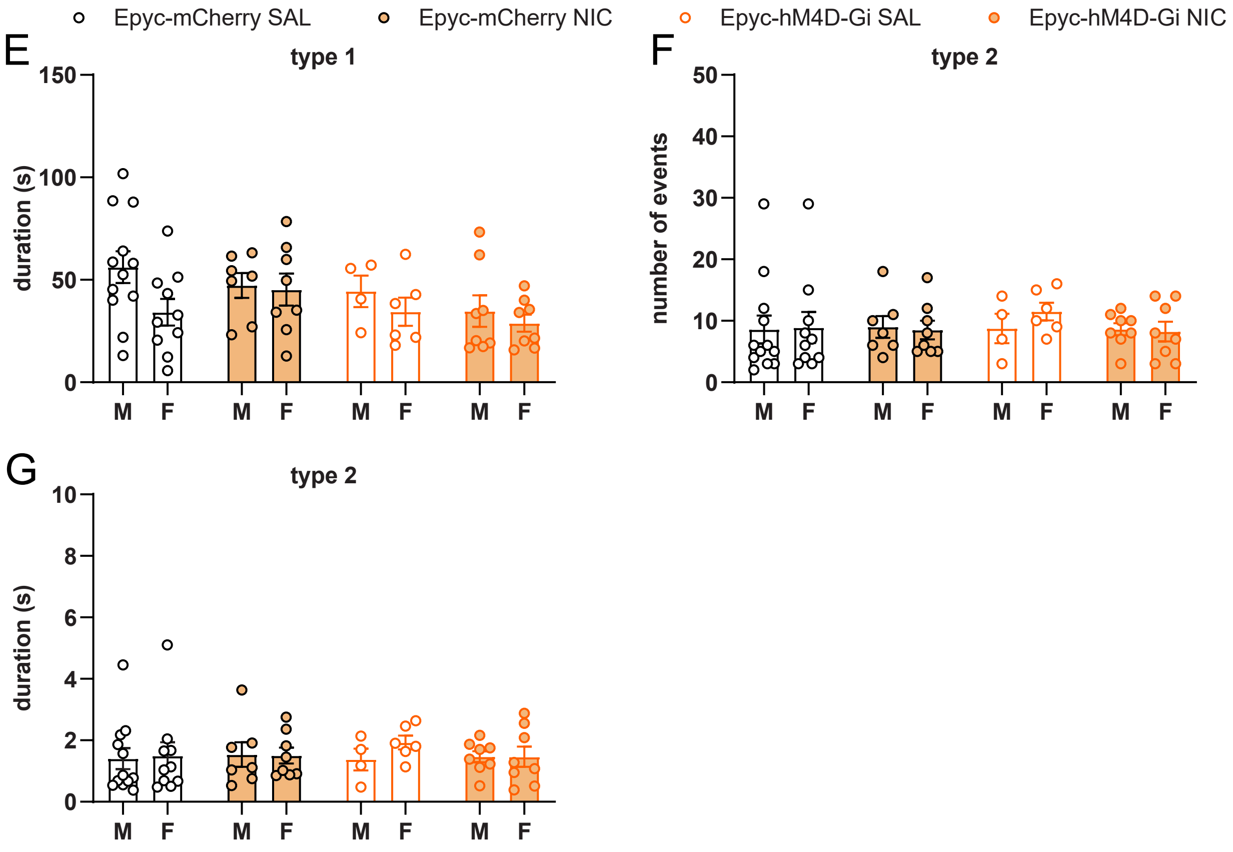

Supplementary Table 4E-G: Three-way ANOVA analysis of main Figure 4 data including sex as a factor

	**E** (re:Figure 4E)		**F** (re:Figure 4F)		**G** (re:Figure 4G)	
Factor	F (DFn, DFd)	p value	F (DFn, DFd)	p value	F (DFn, DFd)	p value
sex	F (1, 55) = 3.41	0.07	F (1, 55) = 0.13	0.72	F (1, 55) = 0.35	0.56
sex x virus	F (1, 55) = 0.15	0.70	F (1, 55) = 0.17	0.68	F (1, 55) = 0.22	0.64
sex x treatment	F (1, 55) = 1.24	0.27	F (1, 55) = 0.41	0.52	F (1, 55) = 0.43	0.52
sex x virus x treatment	F (1, 55) = 0.54	0.47	F (1, 55) = 0.14	0.71	F (1, 55) = 0.17	0.68

Suppl_Figure 15_IPN paper
Miguelez Fernández et al. 2025

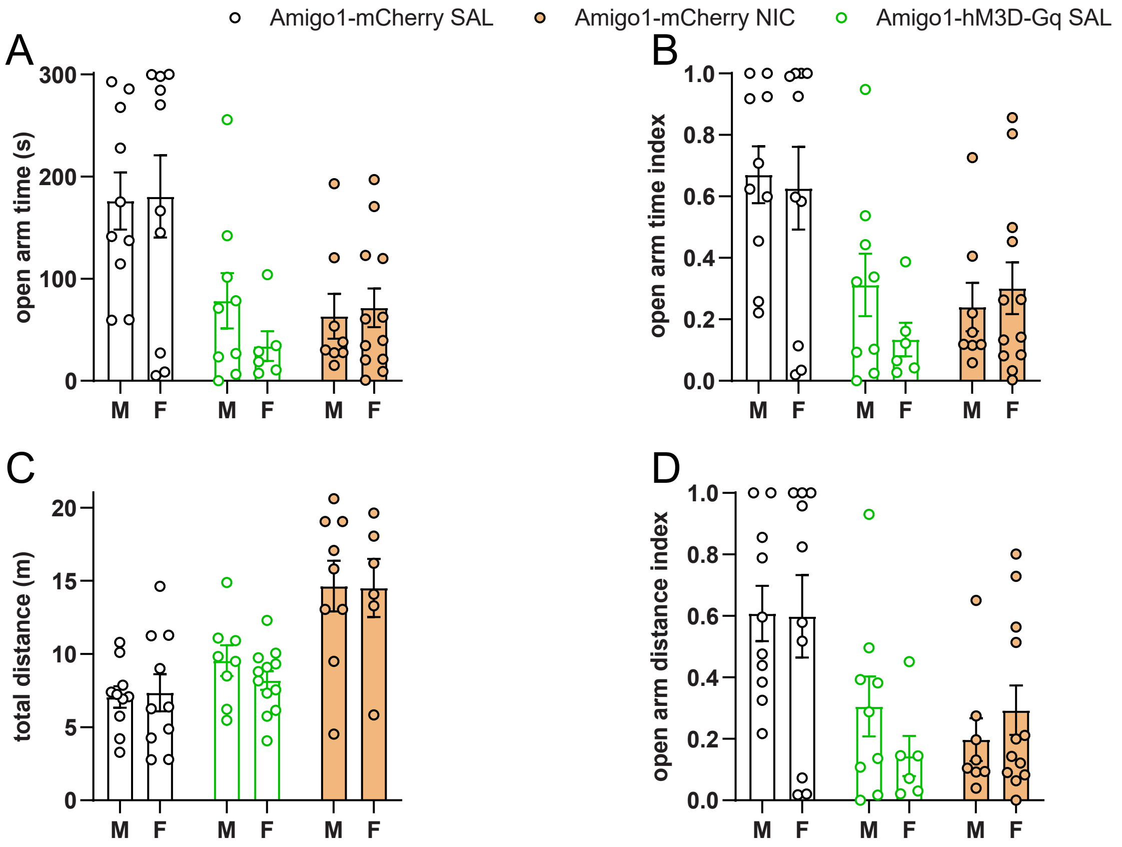

Supplementary Table 5: Two-way ANOVA analysis of main Figure 5 data including sex as a factor

	**A** (re:Figure 5D)		**B** (re:Figure 5E)		**C** (re:Figure 5F)		**D** (re:Figure 5G)	
Factor	F (DFn, DFd)	p value	F (DFn, DFd)	p value	F (DFn, DFd)	p value	F (DFn, DFd)	p value
sex	F (1, 49) = 0.20	0.65	F (1, 49) = 0.41	0.53	F (1, 49) = 0.16	0.69	F (1, 49) = 0.09	0.76
sex x treatment	F (2, 49) = 0.48	0.62	F (2, 49) = 0.64	0.53	F (2, 49) = 0.27	0.77	F (2, 49) = 0.78	0.47

Suppl_Figure 16_IPN paper
Miguelez Fernández et al. 2025

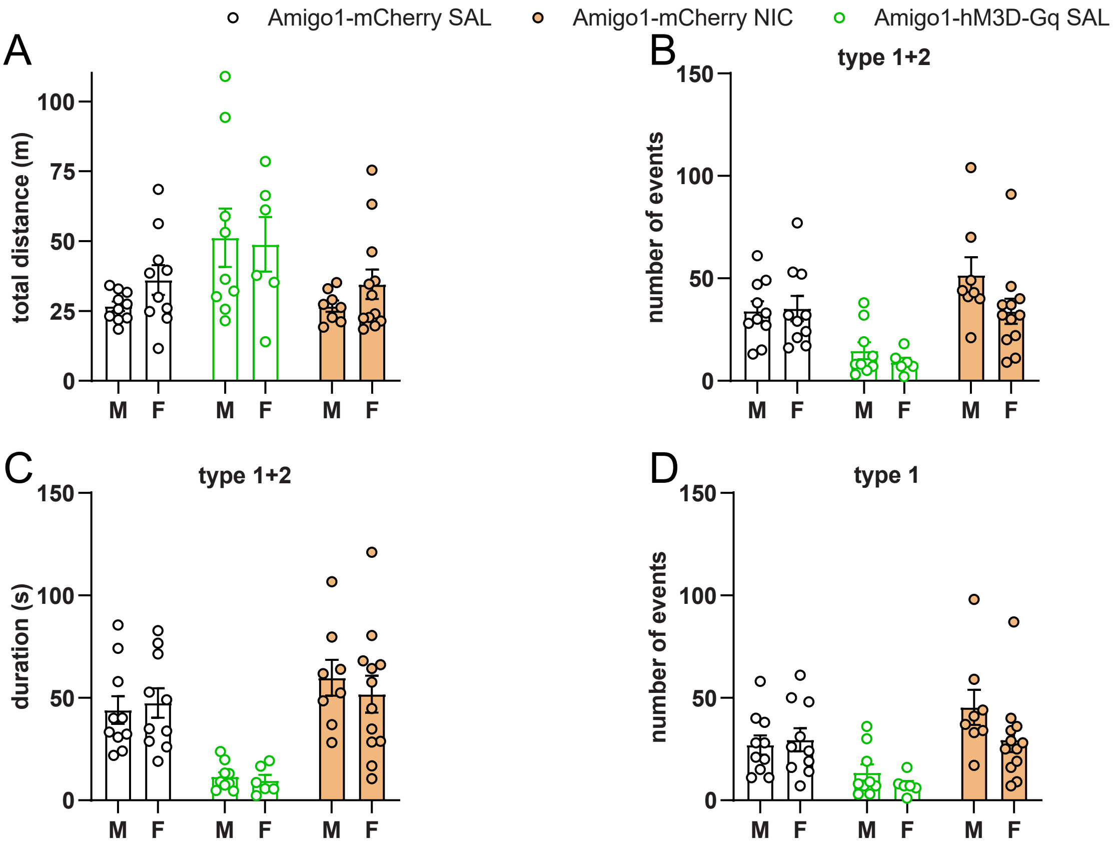

Supplementary Table 6A-D: Two-way ANOVA analysis of main Figure 6 data including sex as a factor

	**A** (re:Figure 6A)		**B** (re:Figure 6B)		**C** (re:Figure 6C)		**D** (re:Figure 6D)	
Factor	F (DFn, DFd)	p value	F (DFn, DFd)	p value	F (DFn, DFd)	p value	F (DFn, DFd)	p value
sex	F (1, 49) = 0.90	0.35	F (1, 49) = 2.14	0.15	F (1, 49) = 0.12	0.73	F (1, 49) = 1.77	0.19
sex x treatment	F (2, 49) = 0.46	0.64	F (2, 49) = 1.33	0.28	F (2, 49) = 0.32	0.73	F (2, 49) = 1.36	0.27

Suppl_Figure 16_IPN paper
Miguelez Fernández et al. 2025

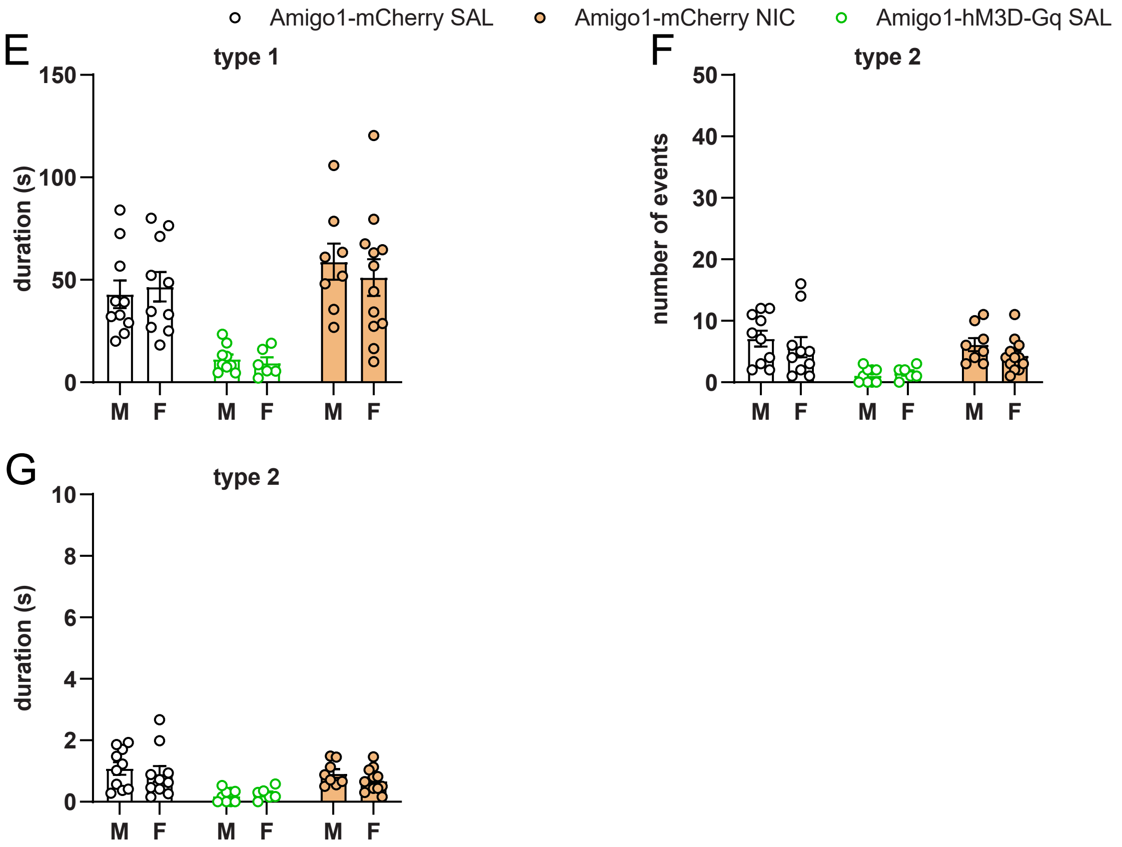

Supplementary Table 6E-G: Two-way ANOVA analysis of main Figure 6 data including sex as a factor

	**E** (re:Figure 6E)		**F** (re:Figure 6F)		**G** (re:Figure 6G)	
Factor	F (DFn, DFd)	p value	F (DFn, DFd)	p value	F (DFn, DFd)	p value
sex	F (1, 49) = 0.11	0.75	F (1, 49) = 1.01	0.32	F (1, 49) = 0.63	0.43
sex x treatment	F (2, 49) = 0.32	0.73	F (2, 49) = 0.47	0.63	F (2, 49) = 0.42	0.66
